# Estimated human intake of endogenous and exogenous hormones from beef in the United States

**DOI:** 10.1038/s41370-024-00727-1

**Published:** 2024-11-07

**Authors:** Ruwan Thilakaratne, Rosemary Castorina, Gina Solomon, Mary M. Mosburg, Benjamin C. Moeller, Josephine F. Trott, Tara D. Falt, Ariadne Villegas-Gomez, Kevin W. Dodd, Catherine Thomsen, Paul English, Xiang Yang, Annika Khan, Asa Bradman, Russell C. Hovey

**Affiliations:** 1 Division of Epidemiology, School of Public Health, University of California, Berkeley, Berkeley, CA USA; 2 Center for Environmental Research and Community Health, School of Public Health, University of California, Berkeley, Berkeley, CA USA; 3 Division of Occupational, Environmental, and Climate Medicine, University of California San Francisco, San Francisco, CA USA; 4 Kenneth L. Maddy Equine Analytical Chemistry Laboratory, School of Veterinary Medicine, University of California, Davis, Davis, CA USA; 5 Department of Molecular Biosciences, School of Veterinary Medicine, University of California, Davis, Davis, CA USA; 6 Department of Animal Science, University of California, Davis, One Shields Ave, Davis, CA USA; 7 Tracking California, Public Health Institute, Oakland, CA USA; 8 Biometry Research Group, Division of Cancer Prevention, National Cancer Institute, Bethesda, MD USA; 9 Zero Breast Cancer, San Rafael, CA USA; 10 Department of Public Health, University of California, Merced, Merced, CA USA

**Keywords:** Melengestrol acetate, Progesterone, Acceptable daily intake

## Abstract

**Background:**

Endogenous and exogenous hormones may be present in beef. Human consumption of hormones has been linked to adverse health effects.

**Objective:**

To estimate daily intake of hormonal growth promotants (HGP) from beef consumed by the US population.

**Methods:**

We combined self-reported beef consumption information from a nationally-representative survey with concentrations of 12 HGP measured in 397 samples of retail beef/fat purchased in California. We defined typical, high, and maximum intake scenarios assuming self-reported consumed beef contained the mean, 95^th^ percentile, and maximum concentrations of each HGP, respectively. We estimated distributions of usual (i.e., long-term) daily intake and short-term daily intake (µg/kg/day). We calculated the hazard quotient (HQ), or ratio of estimated intake to the World Health Organization’s acceptable daily intake (ADI) for the HGP.

**Results:**

The highest estimated HQs were found for melengestrol acetate (MGA). For usual daily intake under the typical intake scenario, no HQ exceeded 0.02 (0.00047 µg MGA/kg/day). Under the maximum intake scenario, the highest HQ was 0.29 (0.0087 µg MGA/kg/day), corresponding to the 99^th^ percentile of intake among young boys (ages 1–5). The highest short-term intake estimates for MGA under the maximum intake scenario were the 99^th^ percentile of intake among young girls and boys, which equaled (HQ = 1.00) or exceeded (HQ = 1.29) the ADI for MGA, respectively.

**Impact:**

- Hormonal growth promotants (HGP) are used to increase beef production and have been linked to adverse reproductive effects. We estimated daily intake of MGA and several other HGP using US nationally-representative beef consumption data collected between 2015–2018 and HGP concentrations in retail beef. Estimated intake was highest for young children, but estimates were generally very low compared to current health-based intake limits. However, these limits are typically based on studies in adult animals, and further study of potential adverse effects during sensitive developmental periods, such as in early life, may be warranted to ensure recommended intake limits are health-protective.

## Introduction

Hormonal growth promotants (HGP) are widely used for the production of beef in the United States (US), both when cattle are in a pre-feedlot growth stage, and when they move into a feedlot for “finishing” to a target weight and body composition. In 2011, a US Department of Agriculture (USDA) survey found approximately 80–90% of feedlots in the US implanted 80–100% of their cattle with HGP during the finishing phase, though notably many feedlots did not participate in the survey, potentially resulting in underestimation of HGP use [1]. In line with this, a more recent survey identified that only 10.7% of feedlots wanted to market their beef as organic or hormone free, suggesting the use of HGP in the beef industry continues to be widespread [2]. Implanted cattle grow 15–20% faster and are 8–12% more efficient at converting feed to beef [1], where HGP are relatively cost-effective and easy to administer.

The US Food and Drug Administration (FDA) authorizes the use of two hormones that are also endogenously produced by beef cattle (17-beta-estradiol [E2], and progesterone [P]) and five synthetic hormones (estradiol benzoate [EB], testosterone propionate [TP], trenbolone acetate [TBA], zeranol [Z], and melengestrol acetate [MGA]) [3]. Estradiol benzoate and Z mimic the anabolic actions of endogenous estrogens, whereas TBA and TP mimic the anabolic actions of endogenous androgens. In contrast, MGA is used to suppress estrus and sexual activity in females (heifers), where it is administered orally via the feed. All other HGP are implanted subcutaneously in the ear either as single agents (i.e., E2 or Z) or in various combinations (TBA + EB, TP + EB, TBA + E2, P + EB) suspended in a silicone or cholesterol matrix for prolonged systemic release. Hormonal growth promotants or their biologically active metabolites may accumulate in muscle and other tissues of the animal, resulting in human intake. For example, EB is cleaved and transformed to E2 in the body, while Z is converted to its active metabolites α- and β-zearalanol. Thus, it is possible that active metabolites could be found in tissues after an animal is treated with an HGP. On the other hand, heifers fed MGA accumulate this HGP at detectable levels in their muscle, fat and liver [4].

The risk for developing certain cancers is influenced by hormone exposure [5]. In the US, as many as 70% of breast cancers may be hormone receptor-positive [6]. Endocrine-disrupting chemicals (EDCs) have long been a significant concern for breast cancer risk, including low doses of EDCs found in plastics, pharmaceuticals, and pesticides [7]. There is also emerging concern regarding the effects of EDCs on pubertal timing, particularly in girls [8]. However, relatively little attention has been given to the potential impact of residual hormones in beef products, despite their frequent and widespread consumption [9, 10], with the average American child consuming approximately 11.6 kg of beef per year [11]. Some have hypothesized that treatment of cattle with HGP could increase the intake of sex steroid hormones by humans, and therefore increase the risk for several diseases, including breast cancer [9, 12, 13]. For example, MGA has been linked to the development of mammary gland tumors in vivo [14, 15].

Published studies measuring HGP residues in beef are limited, and most were conducted outside the US [10, 16–21]. The USDA’s Food Safety Inspection Service National Residue Program (NRP), which commenced in 1967 [22], aims to monitor meat, poultry, and egg products for violative levels of a number of residues, including hormones. However, its sampling plans vary from quarter to quarter with shifting priorities of its collaborating agencies and stakeholders, and, as Nachman and colleagues have observed, the program’s testing for hormone residues has historically been sparse and inconsistent, precluding robust human exposure assessment [9]. The program has collected residue data on only three of the seven HGP, namely TBA, MGA, and Z, and only Z is included in the list of tested residues as of the last update in 2019 [23]. In addition, the annual sampling plan does not specify whether multiple tissues are to be tested from each animal, or whether fat is subsampled as part of muscle sampling [24]. More generally, usual daily dietary intake of HGP from beef has not yet been comprehensively quantified in the US population. For some HGP, the World Health Organization (WHO) has established acceptable daily intakes (ADIs), or the maximum daily intake over a lifetime that is believed not to cause adverse health consequences based on existing toxicological studies, to which estimates of daily HGP intake could be compared to assess potential public health risk [25].

In this study, we aimed to provide an initial assessment of human intake of HGP from beef in the US. We measured various endogenous and exogenous hormones in hundreds of retail beef products, spanning a variety of meat cuts and organs that were sourced across the state of California. We combined these data with nationally-representative dietary recall data and statistical modeling to estimate distributions of usual (i.e., long-term) daily intake of HGP (µg HGP/kg bodyweight/day), and then compared these with current WHO recommendations for intake limits. We estimated sex-specific intake across life stages, including three subgroups for children (early childhood, ages 1–5; middle childhood, ages 6–9; adolescence, ages 10–19) and two for adults (young-to-middle adulthood, ages 20–54; late adulthood or post-menopausal, ages 55+). Additionally, we compared potential intake levels between subgroups defined by race/ethnicity, income, and education level, to identify potential socioeconomic disparities in HGP intake.

## Methods

### Dietary recall data from the National Health and Nutrition Examination Survey

We used publicly accessible dietary recall data from the 2015–2016 and 2017–2018 cycles of the National Health and Nutrition Examination Survey (NHANES) [26, 27] to describe beef consumption by US females and males one year of age and older. The NHANES program monitors the health and nutritional status of the non-institutionalized US population by implementing a nationally representative survey of approximately 10,000 individuals in two-year cycles. Details regarding survey design and data collection are available online (https://www.cdc.gov/nchs/nhanes/index.htm). Dietary intake was assessed by two 24-h recalls (24HR). At the in-person recruitment visit, participants completed the first 24HR, in which they were asked to recall foods and beverages consumed in the previous 24-h period, as well as their respective quantities, which were converted to gram amounts. A second 24HR was solicited by telephone 3–10 days later.

### Estimating raw beef intake from consumed beef-containing foods

To estimate beef intake in NHANES, we followed the approaches of previous studies [11, 28] with several modifications to derive HGP intake from raw beef intake. NHANES assigns each food an 8-digit numeric code (“food code”) generated by the United States Department of Agriculture (USDA) in its Food and Nutrient Database for Dietary Studies (FNDDS) [29]. For each food, the FNDDS provides a general description, a standard set of ingredients and their relative proportions (“recipes”), and nutrient values. Recipes were generated by USDA using multiple data sources, including USDA food product databases, company websites, product preparation instructions, label ingredients, cookbooks, and recipe websites. Each recipe provides the weight in grams of each ingredient, and aims to represent several possible variants of each food. To ensure beef ingredients used in multi-ingredient foods were included, we disaggregated all foods into constituent ingredients by linking food codes to ingredient codes using FNDDS. Beef ingredients were defined as ingredients containing the word “beef” in their description. All beef-containing foods and their beef ingredients are provided in Supplementary Table S1.

When beef was an ingredient in a recipe, we calculated grams of beef consumed by multiplying the consumed weight (in grams) by the fraction of the total weight of the food’s recipe that was comprised of the beef ingredient(s). Raw weight of beef was estimated, for later linkage with HGP concentrations measured in raw retail beef products, by dividing the beef ingredient’s cooked weight by a moisture adjustment factor of 0.75 (assuming 25% of weight is lost as water during cooking). This factor is used by FNDDS to account for moisture loss during cooking when determining nutritional value [30, 31]. The steps are described in Eq. (1) below.

1 $${{{\rm{raw}}}}\; {{{\rm{beef}}}}\left({{{\rm{g}}}}\right)= 	 \, {{{\rm{beef}}}}-{{{\rm{containing\; food}}}}\left({{{\rm{g}}}}\right) \\ 	 \times{{{\rm{beef}}}} \; {{{\rm{fraction}}}}\div{{{\rm{moisture}}}}\; {{{\rm{adjustment}}}}\; {{{\rm{factor}}}}$$

Where beef fraction is the proportion of the food, by weight, comprised of beef ingredients, and the moisture adjustment factor to account for moisture loss (to convert cooked beef to raw beef) is 0.75.

### Measurement of HGP concentrations in retail beef

Retail cuts of beef were purchased from four regions in California, namely the San Francisco Bay Area (San Francisco and Alameda Counties), Central Valley (Fresno and Merced Counties), Salinas Valley (Monterey County), and Southern California (Los Angeles and Riverside Counties). The Bay Area and Southern California are urban centers that contain the majority of the state’s population. The Central Valley and Salinas Valley are agricultural areas inhabited by a predominately Hispanic population that has culturally- and socioeconomically-diverse dietary patterns and associated beef sourcing when compared to other areas.

We classified retailers into three types: traditional retail chains (e.g., supermarket chains, supercenters), specialty stores (e.g., ethnic grocers, specialty butchers), and limited assortment retailers (e.g., discount, warehouse). Because much of the sampling was done during the COVID-19 pandemic, we generally limited the geographic spread to allow travel to and between sites within one day. Some products were sampled in San Francisco County over two days in May 2020, in Alameda County over five days in July 2020, in Riverside County over two days in September 2020, in Fresno County on one day in May 2021, in Los Angeles and Orange Counties over two days in March 2022, and in Salinas on one day in May 2022. For a subset of larger counties (Riverside, Fresno, Alameda, and San Francisco) each retailer in each county was assigned a number and one retailer was randomly selected for each of the 3 types of retailers using the online Research Randomizer tool (https://www.randomizer.org). A fourth retailer in each county was randomly selected from all retailers. For regions where more than four retailers were surveyed (e.g. Fresno, Alameda), two retailers (one Latinx and one Asian) were randomly selected from the ethnic/specialty category for sampling. For Orange, Los Angeles and Salinas Counties, retailers of all three types were randomly selected. In total, 59 unique retailers were visited for the purpose of purchasing retail beef products.

Given that over 140 different beef cuts are sold at retail, we chose to purchase a subset of priority cuts to account for various characteristics including fat *versus* lean, affordability, cuts and organs available at ethnic grocers, and top 10 cuts by sales in California. We aimed to buy beef cuts from each of six categories from each store, if available. These categories were ground beef of high or low fat content, chuck, loin, round, and organs (e.g., liver, heart, tripe). Some products included common marketing claims, including organic, hormone-free, or grass-fed, while others had no marketing claims. All label information and marketing claims were recorded at the time of purchase, and samples photographed. All samples (*n* = 321), in their original packaging, were placed into pre-labeled plastic bags to prevent cross-contamination, and were initially kept frozen on dry ice until they were transported back to the lab for storage at −20 °C. To account for potential differential accumulation of HGP in adipose tissue *versus* other tissues [4] for estimating HGP intake, visible subcutaneous fat was separated from *n* = 76 retail beef products and analyzed separately. Ground beef was only analyzed as the composite of meat and adipose. We did not account for differences in HGP concentrations by beef cut (i.e., chuck, round, loin, etc.) due to insufficient sample size to detect statistically significant differences in HGP concentrations between cuts, and insufficient resolution in the NHANES dietary recall data to distinguish the precise beef cuts consumed, whereas fat *versus* other components of beef are distinguishable in NHANES using FNDDS nutritional information.

All muscle, fat and organ samples were processed as described using a methodology that was validated for bovine muscle samples [32]. Briefly, a 100 ± 5 mg piece of frozen tissue was weighed and placed into 7 mL bead beating tubes containing 2.8 mm ceramic beads (Bertin Corp, Rockland, MD) and 4 mL of 0.1 M phosphate buffer, pH 9.5. Samples were homogenized using a bead beater, then extracted with a 70:30 (v:v) mixture of hexanes and ethyl acetate prior to drying and reconstitution in 80:20 (v:v) methanol:0.2 mM ammonium fluoride. Extracts were then analyzed for 12 endogenous or synthetic HGP or their esterified version, namely estradiol, E2, EB, melengestrol, MGA, P, epitestosterone (EpiT), testosterone (T), TP, trenbolone (TB), TBA, and α-zearalanol. Samples were analyzed by ultra-high performance liquid chromatography triple quadrupole-mass spectrometry (UHPLC-MS/MS) system consisting of a Thermo Vanquish Duo UHPLC, combined with a Thermo Altis TSQ MS (ThermoFisher Scientific, Waltham, Massachusetts, USA). The method was validated for accuracy, precision, recovery, matrix effects, limits of detection, limits of quantitation, linear range and stability [32].

We estimated intake for HGP that were detected in at least ten beef samples. For these HGP, we imputed non-detects as the limit of detection (LOD) divided by the square root of two, prior to performing data analyses [33]. These HGP (LOD, number of samples with a detection) included MGA (0.1 pg/mg, *n* = 78), P (0.1 pg/mg, *n* = 93), T (0.1 pg/mg, *n* = 22), and EpiT (0.1 pg/mg, *n* = 82). We excluded those HGP with fewer or no detections, which included trenbolone (*n* = 1 detection), E2 (*n* = 0), estradiol (*n* = 0), EB (*n* = 0), melengestrol (*n* = 0), TP (*n* = 0), TBA (*n* = 0), and α-Z (*n* = 0).

### Assumed HGP concentrations in consumed beef: intake scenarios

We specified three intake scenarios, namely “typical”, “high”, and “maximum” (hereinafter, “max”), to represent assumptions about the extrapolation of measured HGP concentrations in retail beef to those in beef consumed by NHANES participants. The typical intake scenario uses the average concentration of each HGP; the high intake scenario uses the 95^th^ percentiles; and the max scenario uses the highest measured concentrations. These scenarios represent intake that would occur if the consumed beef contained: HGP concentrations consistent with the observed distribution in the retail samples, after imputation of non-detects (“typical” intake scenario); high concentrations of HGP (“high” intake scenario); or the maximum observed concentration of HGP (“max” intake scenario). The typical intake scenario is specified to be most representative of typical HGP intake for the US population, assuming that the concentration distribution in our beef samples is representative of the US beef supply. While the high and max intake scenarios are unlikely to occur US-wide, the high intake scenario may estimate intake by individuals whose beef is sourced from cattle having above-average concentrations of HGP (i.e., the 95^th^ percentile of the US beef supply). The max intake scenario aims to establish the upper bound of HGP intake.

### Estimating single-day HGP intake using 24HRs

To calculate per-bodyweight HGP intake for a single 24HR for a given NHANES participant, we first calculated fat and non-fat raw beef intakes (g) for a given beef ingredient by multiplying the ingredient amount by its fat proportion and by the remaining non-fat proportion, respectively. For simplicity, we use the term “non-fat” to collectively refer to all samples other than the visible subcutaneous fat subsamples (i.e., muscle, ground meat, and organs), but we note that these other samples almost certainly contain some amount of fat that was not easily separable from the rest of the muscle or other tissue. Fat proportions were calculated from the FNDDS nutrition label for each ingredient as the total grams of fat divided by the total grams in the ingredient. Subsequently, these intakes were multiplied by fat and non-fat HGP concentrations (pg/mg), respectively, as assumed under each of the three intake scenarios. Intakes of HGP were then summed across all beef ingredients consumed within the individual’s 24HR, and across fat and non-fat intakes, to obtain total HGP intake. Finally, this quantity was divided by the individual’s bodyweight measured in NHANES, and a scaling factor of 1000, to determine µg HGP/kg bodyweight/day.

2 $${{{\rm{HGP}}}}_{{is}}= 	 {\sum}_{j=1}^{k}\left({{{\rm{raw}}}}\, {{{\rm{beef}}}}_{{ij}}({{\rm{g}}})\times {{\rm{fat}}}\, {{{\rm{proportion}}}}_{j} \times {{{\rm{HGP}}}}_{{{\rm{fat}}},s}({{\rm{pg}}}/{{\rm{mg}}})\right. \\ 	 + \left. {{\rm{raw}}}\, {{{\rm{beef}}}}_{{ij}}({{\rm{g}}})\times (1-{{\rm{fat}}}\, {{{\rm{proportion}}}}_{j})\times {{{\rm{HGP}}}}_{{{\rm{non}}}-{{\rm{fat}}},s}({{\rm{pg}}}/{{\rm{mg}}})\right)\\ 	 \div\left(1000\times {{\rm{bodyweight}}}({{\rm{kg}}})\right)$$

Where HG*P*_*is*_(μg/kg/day) is the estimated total HGP per kg bodyweight consumed by participant $i$ on a given day under intake scenario $s$ (typical, high, or max); raw ${{\rm{beef}}}_{ij}({{\rm{g}}})$ is the amount of beef ingredient $j$ reported consumed by participant $i$; fat proportion is the fraction of the beef ingredient $j$ comprised of fat according to the ingredient’s nutrient information; HGP_fat,*s*_ is the HGP concentration in fat for intake scenario $s$; and HGP_non-fat,*s*_ is the HGP concentration in non-fat for intake scenario $s$. The HGP amounts were summed across the $j={{\mathrm{1,2}}},\ldots ,k$ beef ingredients that were reported consumed by participant $i$ on a given day.

### Estimating usual daily intake of HGP

We employed the National Cancer Institute (NCI) method [34, 35] to estimate the distribution of usual (i.e., long-term) daily HGP intake. Using a limited number of short-term dietary assessments, the NCI method leverages statistical modeling to account for random within-person measurement error. Notably, this method cannot account for systematic error (e.g., errors or biases in self-report) that is likely present in the 24HR. To do so would require an additional calibrating assessment method that is known to be free of systematic error, which is not available for beef consumption in NHANES. The NCI method can be used for episodically consumed foods or other substances, such as HGP from beef, when at least one 24HR is available for all individuals, and at least two 24HRs are available for a subset of the population, as is the case in NHANES. The NCI method uses a two-part mixed effects model. In the first part, the “probability model”, a logistic mixed effects regression model is fitted, assuming the participant-level probability of HGP consumption on a given day is a function of a group-level baseline probability that may be influenced by one or more covariates, and a normally-distributed person-specific random intercept that represents a deviation from the group-level baseline. In the second part, the “amount model”, a linear mixed effects model for the amount of HGP consumed on consumption days (i.e., days when the amount of HGP consumed is >0), is fitted, assuming a similar function of a group-level, potentially covariate-influenced mean, and a normally-distributed, person-specific random intercept. We allowed the two parts to be correlated as the probability of beef consumption was positively correlated with the usual amount of beef consumed on consumption days [34]. Separate models were run for children (ages 1–19) and adults (ages 20+), with correlation between probability and amount models permitted for both. Sampling weights provided by NHANES accounted for the complex survey design, non-response to either 24HR, and increased responsiveness on weekends. Sampling weights were used in all models and analyses to generate population-representative estimates.

The probability model was specified as follows:

3 $${logit}\left(P\left({R}_{{ij}}\, > \, 0|i\right)\right)={\beta }_{10}+{{{{\boldsymbol{\beta }}}}}_{{{{{\boldsymbol{X}}}}}_{{{{\bf{1}}}}}}{{{{\boldsymbol{X}}}}}_{{{{\bf{1}}}}{{{\boldsymbol{ij}}}}}+{u}_{1i}$$

Where ${R}_{{ij}}$ is the consumption amount for person $i={{\mathrm{1,2}}},\ldots ,n$ for the $n$ participants, and consumption day $j\in \{{{\mathrm{1,2}}}\}$; ${{{{\rm{\beta }}}}}_{10}$ is the grand intercept; ${{{{\boldsymbol{\beta }}}}}_{{{{{\boldsymbol{X}}}}}_{{{{\boldsymbol{1}}}}}}$ is a vector of coefficients for each element of the covariate vector ${{{{\boldsymbol{X}}}}}_{{{{\boldsymbol{1}}}}{{{\boldsymbol{ij}}}}}$; and ${u}_{1i}$ is a person-specific random intercept, where ${u}_{1i}\sim {Normal}(0,{{{{\rm{\sigma }}}}}_{{u}_{1}}^{2})$. ${{{{\boldsymbol{X}}}}}_{{{{\boldsymbol{1}}}}{{{\boldsymbol{ij}}}}}$ contains the a priori selected covariates of self-reported gender (male or female); age (continuous); a linear spline for age with a knot at 11 years for the children, and at 29, 39, 49, 59, and 69 for adults, following NHANES 2015–2018 sampling design [36]; cross-product terms between age variables and gender to allow age effects to vary by gender; an indicator for whether the 24HR took place at the end of the week (Friday, Saturday, or Sunday) or during the week (Monday through Thursday); and an indicator for the first 24HR (in-person) or second 24HR (by phone).

The amount model was specified as follows:

4 $$g({R}_{{ij}} | {R}_{{ij}}\, > \, 0 \, ; \, \lambda )={{{{\rm{\beta }}}}}_{20}+{{{{\boldsymbol{\beta }}}}}_{{{{{\boldsymbol{X}}}}}_{{{{\bf{2}}}}}}{{{{\boldsymbol{X}}}}}_{{{{\bf{2}}}}{{{\boldsymbol{ij}}}}}+{u}_{2i}+{{{{\rm{\epsilon }}}}}_{{ij}}$$

Where ${{{{\rm{\beta }}}}}_{20}$ is the grand intercept; ${{{{\boldsymbol{\beta }}}}}_{{{{{\boldsymbol{X}}}}}_{{{{\boldsymbol{2}}}}}}$ is a vector of coefficients for each element of the covariate vector ${{{{\boldsymbol{X}}}}}_{{{{\boldsymbol{2}}}}{{{\boldsymbol{ij}}}}}$; ${u}_{2i}$ is a person-specific random intercept, where ${u}_{2i}\sim N{{{\rm{ormal}}}}(0,{{{{\rm{\sigma }}}}}_{{{{{\rm{u}}}}}_{2}}^{2})$; ${{{{\rm{\epsilon }}}}}_{{ij}}$ is the noise term, where ${{{{\rm{\epsilon }}}}}_{{ij}}\sim {Normal}\left(0,{{{{\rm{\sigma }}}}}^{2}\right)$; and $g({R}_{{ij}}|{R}_{{ij}}\, > \,0;\lambda )$ is the Box-Cox transformation with parameter λ that is applied to reported intakes ${R}_{{ij}}$ on consumption days to achieve approximate normality of the ${u}_{2{{{\rm{i}}}}}$ and ${{{{\rm{\epsilon }}}}}_{{{{\rm{ij}}}}}$ terms [34]. ${{{{\boldsymbol{X}}}}}_{{{{\boldsymbol{2}}}}{{{\boldsymbol{ij}}}}}$ contains a priori covariates identical to those specified as ${X}_{1{ij}}$ in the probability model (i.e., gender, age, age spline terms, age-gender cross-product terms, end-of-week, and 24HR sequence).

Fitted models were used to simulate 100 usual intake estimates on the original scale (after adjusting for the Box-Cox transformation and the noise term) for each person, representing a sample of “pseudo-persons” that accounts for the distribution of covariates in the population. We aggregated simulated intakes according to prespecified gender- and age-specific groupings based on anticipated changes in dietary patterns with age, and susceptible periods. For females, groups were defined as early childhood (1–5 years), middle childhood (6–9 years), adolescence (10–19 years), young-to-middle adulthood (20–54 years), and postmenopausal (55+). For males, groups were defined as early childhood (1–5 years), middle childhood (6–9 years), adolescence (10–19 years), young-to-middle adulthood (20–54 years), and late adulthood (55+). All groupings had at least 50 individuals reporting consumption of beef on both 24HRs, the recommended minimum sample size to achieve sufficiently stable models for intake simulations. We reported the mean, median, 95^th^ percentile, and 99^th^ percentile of the estimated distribution of intake for each HGP for each grouping, under each of the three intake scenarios.

### Comparison of daily HGP intake estimates to acceptable daily intakes (ADIs)

We estimated distributions of long-term daily HGP intake to enable comparison to ADIs established by the Joint Food and Agriculture Organization of the United Nations/WHO Expert Committee on Food Additives (JECFA) for MGA [14], T [37], and P [38]. We estimated T intake as the sum of T and EpiT intakes, and compared this sum to the ADI for T. The ADIs express the maximum daily per-bodyweight intake (µg/kg/day) in humans that, over a lifetime of consumption, is not expected to result in adverse health effects. ADIs are established using toxicological studies that identify no observed effect levels (NOELs) or lowest observed effect levels (LOELs) in animals or humans, to which a so-called “safety factor” may be applied that lowers the ADI to ensure human health is protected. For a given compound, there is a single ADI for humans, regardless of age, gender, or other factors. The ADIs for the HGP under examination herein are based on studies with endocrine or reproductive endpoints, and are described in Supplementary Table S2. The hazard quotient (HQ) for an estimated mean or percentile of usual intake was calculated by dividing estimated intake by the ADI, where an HQ above 1 indicates the estimated intake exceeds the ADI. A chronic hazard index (HI) for the joint usual daily intake of MGA, progesterone, and testosterone, which would be the sum of their HQs, may be appropriate given their critical effects are all roughly related to the reproductive system (Supplementary Table S2) [1]. However, the HQs we estimated for usual daily intake of progesterone and testosterone were negligible (all <0.01), particularly compared to those for MGA (e.g., ranging from 0.04 to 0.29 under the max intake scenario), and therefore we did not separately calculate chronic HIs, as they would be approximately equal to the HQs for MGA.

As a supplementary analysis, we also calculated empirical distributions of the two-day average reported HGP intake among individuals with two 24HRs, and report the same percentiles of intake and HQs for these distributions. Because these short-term intakes are not strictly comparable to chronic ADIs, we did not calculate acute HIs.

### Sociodemographic determinants of HGP intake

Using sociodemographic variables as covariates in NCI models, we estimated ratios of median usual daily MGA intake under the typical intake scenario, comparing strata of several social determinants of health (race/ethnicity, poverty, and education) to the intake of the overall population. We used the median of the intake distribution and typical intake scenario to make the results more representative of the average US individual, and focused on MGA because estimated usual intake means and percentiles were highest for this HGP relative to its ADI. We conducted analyses separately among all children (ages 1–19) and adult women (ages 20+) given that the ADI for MGA is based on its ability to alter endocrine function and menstrual cyclicity, which may affect puberty onset [39] and reproduction [40], thus specifically impacting children and women. We did not further stratify into age subgroups due to insufficient sample sizes to support NCI method modeling. For children, intake ratios were calculated for race/ethnicity (non-Hispanic Asian, non-Hispanic Black, Mexican American/other Hispanic, other race/multi-racial, and non-Hispanic White, *versus* overall child intake), and annual household income relative to the poverty line as defined using the 2015–2016 and 2017–2018 Department of Health and Human Services’ poverty guidelines, categorized into two groups (≤130% of the federal poverty line (FPL) and >130% of the FPL, *versus* overall child intake). For adult women, in addition to race/ethnicity and poverty threshold, we also estimated intake ratios for each of two education levels (high school/General Education Development diploma or less, and some college or above, *versus* overall adult women intake). To estimate 95% confidence intervals (CIs) for the intake ratios, we used balanced repeated replication (BRR), a method for estimating sampling variability in complex surveys, described in detail elsewhere [41]. We used 32 BRR replicates with Fay’s method (F = 0.3), as recommended in previous work when using the NCI method on two cycles of NHANES dietary recall data [41]. For women’s education, two replicates failed to converge and therefore 30 BRR replicates were used [41].

We set α = 0.05 for a two-sided hypothesis test to define statistical significance. Comparison of HGP levels in paired meat and fat samples was performed using a Wilcoxon matched-pairs signed rank test in Prism 10.0 (GraphPad Software, Boston, Massachusetts, USA). Statistical analyses using the NCI method were performed in SAS version 9.4 (SAS Institute Inc., Cary, North Carolina, USA), and analyses for two-day averages and visualizations were generated in R version 4.2.3 [42].

## Results

### Retail beef samples and concentrations

In total, 321 beef products were purchased from across California. Of these, 76 were subsampled for their fat to realize a total of 397 unique samples, which were analyzed for HGP. The largest share of beef products was from Southern California (45.8%), followed by Salinas (33%), the Bay Area (13.1%), and the Central Valley (8.1%) (Supplementary Table S3). The most frequently analyzed component/cut of beef was sub-sampled fat tissue (19.1%), followed by chuck (15.1%), ground beef with 11–20% fat (10.3%), and ground beef with 0–10% fat (10.1%). Several organ meats were also analyzed, such as liver (6.0%), stomach (6%), heart (1.3%), and tongue (0.5%) (Supplementary Table S4). A minority of samples came from products labeled as USDA choice (9.7%), hormone-free (9.7%), organic (5.3%), or grass-fed (6.2%) (Supplementary Table S5). A majority (59.2%) of the 321 samples were purchased at a butcher counter and either wrapped in paper or placed in a plastic bag. A USDA (or Uruguay) inspection seal was present on 20.9% of our samples. Unfortunately, some (*n* = 13) establishment codes were not found and recorded before the packaging was discarded. The 54 establishment codes we recorded encompassed 22 different establishments located across California (51.9%), Arizona (16.7%), Oregon (7.4%), New Jersey (7.4%), Utah (3.7%), Montana (3.7%), Nevada (3.7%), Kansas (1.85%), Iowa (1.85%) and Uruguay (1.85%). Limits of detection, detection frequencies, and distribution statistics for the five HGP with at least one detection across fat or non-fat samples are provided in Table 1. The HGP detection frequencies among the 76 fat samples were highest for EpiT (36%) followed by MGA (25%), P (21%), and T (4%). Detection rates in the 321 non-fat beef samples were highest for P (24%) followed by MGA (18%), EpiT (17%), and T (6%). Only one sample, a fat sample, tested positive for trenbolone. Average concentrations of P (*p* = 0.02), MGA (*p* < 0.01), T (*p* = 0.375), and EpiT (*p* < 0.01) were higher in fat compared to the adjacent meat. For example, the mean (95^th^ percentile) EpiT concentrations were 0.20 (0.61) pg/mg in fat and 0.10 (0.26) pg/mg in non-fat.

**Table 1:** Detection rates and concentration distributions for each of five hormones, by fat tissue and non-fat (muscle, organs, ground, etc.) tissue.

Tissue	HGP	LOD (pg/mg)	*N* samples	Percent detected (*N*)	Mean (pg/mg)	95th percentile (pg/mg)	Max (pg/mg)
Fat	Epitestosterone	0.1	76	36% (27)	0.20	0.61	3.09
	Melengestrol Acetate	0.1	76	25% (19)	0.65	3.25	4.07
	Progesterone	0.5	76	21% (16)	4.91	48.20	70.10
	Testosterone	0.1	76	4% (3)	0.20	0.07^a^	4.91
	Trenbolone	0.1	76	1% (1)	0.07	0.07^a^	0.16
Non-fat	Epitestosterone	0.1	321	17% (55)	0.10	0.26	1.07
	Melengestrol Acetate	0.1	321	18% (59)	0.13	0.40	2.38
	Progesterone	0.5	321	24% (77)	1.84	9.41	26.40
	Testosterone	0.1	321	6% (19)	0.09	0.15	1.38
	Trenbolone	0.1	321	0% (0)	0.07^a^	0.07^a^	0.07^a^

Samples below the limit of detection were imputed as the limit of detection divided by the square-root of 2, prior to calculation of distribution statistics.

*HGP* hormonal growth promotant, *LOD* limit of detection.

^a^Equivalent to the limit of detection divided by the square root of 2.

### Estimated usual daily intake distributions based on the NCI method

We estimated usual daily intake for MGA, T, and P, given that these HGP were detected in at least 10 samples. Selected characteristics of the estimated usual MGA intake distribution (µg/kg/day) for male and female children and adults are provided in Table 2. Estimated intake tended to decrease with increasing age among males, while among females, estimated intake decreased from childhood to adulthood but increased in the postmenopausal period. In general, estimated distributions of intake were higher for males than females. No characteristics of the estimated distribution of usual daily intake of MGA exceeded its ADI of 0.03 µg/kg/day for any of the subgroups under any intake scenario. The highest estimated MGA intake (99^th^ percentile, max intake scenario) was for males in early childhood (ages 1–5) with 0.0087 µg/kg/day, corresponding to an HQ of 0.29. The estimated median usual intake under the typical intake scenario, which aims to represent the intake of the typical US individual, was also highest for males in early childhood (ages 1–5) with 0.00018 µg/kg/day, corresponding to an HQ of 0.01. Estimated distributions of usual daily P intake are presented in Table 3. None of the estimated usual daily intake distribution for P exceeded the ADI of 30 µg/kg/day [38]. The highest estimate of usual daily P intake (99^th^ percentile, max intake scenario) was for males in early childhood (ages 1–5), with 0.098 µg/kg/day, corresponding to an HQ of <0.01. The highest estimated median intake for P under the typical intake scenario was 0.0027 µg/kg/day among males in early childhood (ages 1–5), corresponding to an HQ of <0.01.

**Table 2:** Estimates of usual daily melengestrol acetate intake from beef consumption across various demographic groups and intake scenarios in a nationally representative US sample from the National Health and Nutrition Examination Survey, 2015–2018.

Demographic category (age range in years)			Melengestrol acetate intake from estimated usual daily intake^ab^ of beef, µg/kg/day (Hazard quotient)^c^				
	Total *n*^d^	Eaters *n*^e^	Mean	Median	95th%	99th%	ADI (µg/kg/day)
Typical intake scenario^f^							
Females							
Early childhood (1–5)	798	120	0.00014 (<0.01)	0.00013 (<0.01)	0.00026 (0.01)	0.00033 (0.01)	0.03
Middle childhood (6–9)	605	84	0.0001 (<0.01)	0.000095 (<0.01)	0.0002 (0.01)	0.00027 (0.01)	0.03
Adolescence (10–19)	1430	220	0.000071 (<0.01)	0.000064 (<0.01)	0.00014 (<0.01)	0.00019 (0.01)	0.03
Young-to-middle adulthood (20–54)	2883	410	0.000082 (<0.01)	0.000078 (<0.01)	0.00014 (<0.01)	0.00017 (0.01)	0.03
Postmenopausal (55+)	2154	350	0.0001 (<0.01)	0.000099 (<0.01)	0.00018 (0.01)	0.00022 (0.01)	0.03
Males							
Early childhood (1–5)	823	120	0.0002 (0.01)	0.00018 (0.01)	0.00036 (0.01)	0.00047 (0.02)	0.03
Middle childhood (6–9)	612	94	0.00018 (0.01)	0.00017 (0.01)	0.00034 (0.01)	0.00045 (0.01)	0.03
Adolescence (10–19)	1428	210	0.00015 (<0.01)	0.00013 (<0.01)	0.00029 (0.01)	0.00038 (0.01)	0.03
Young-to-middle adulthood (20–54)	2576	390	0.00014 (<0.01)	0.00013 (<0.01)	0.00023 (0.01)	0.00028 (0.01)	0.03
Late adulthood (55+)	2146	340	0.00013 (<0.01)	0.00012 (<0.01)	0.0002 (0.01)	0.00024 (0.01)	0.03
High intake scenario^f^							
Females							
Early childhood (1–5)	798	120	0.00044 (0.01)	0.00041 (0.01)	0.00082 (0.03)	0.001 (0.03)	0.03
Middle childhood (6–9)	605	84	0.00033 (0.01)	0.0003 (0.01)	0.00063 (0.02)	0.00084 (0.03)	0.03
Adolescence (10–19)	1430	220	0.00022 (0.01)	0.0002 (0.01)	0.00044 (0.01)	0.0006 (0.02)	0.03
Young-to-middle adulthood (20–54)	2883	410	0.00026 (0.01)	0.00024 (0.01)	0.00043 (0.01)	0.00053 (0.02)	0.03
Postmenopausal (55+)	2154	350	0.00032 (0.01)	0.00031 (0.01)	0.00055 (0.02)	0.00068 (0.02)	0.03
Males							
Early childhood (1–5)	823	120	0.00062 (0.02)	0.00057 (0.02)	0.0011 (0.04)	0.0015 (0.05)	0.03
Middle childhood (6–9)	612	94	0.00057 (0.02)	0.00053 (0.02)	0.0011 (0.04)	0.0014 (0.05)	0.03
Adolescence (10–19)	1428	210	0.00046 (0.02)	0.00042 (0.01)	0.00089 (0.03)	0.0012 (0.04)	0.03
Young-to-middle adulthood (20–54)	2576	390	0.00043 (0.01)	0.00041 (0.01)	0.00073 (0.02)	0.00087 (0.03)	0.03
Late adulthood (55+)	2146	340	0.00039 (0.01)	0.00038 (0.01)	0.00064 (0.02)	0.00076 (0.03)	0.03
Max intake scenario^f^							
Females							
Early childhood (1–5)	798	120	0.0026 (0.09)	0.0024 (0.08)	0.0048 (0.16)	0.0062 (0.21)	0.03
Middle childhood (6–9)	605	84	0.0019 (0.06)	0.0018 (0.06)	0.0037 (0.12)	0.005 (0.17)	0.03
Adolescence (10–19)	1430	220	0.0013 (0.04)	0.0012 (0.04)	0.0026 (0.09)	0.0036 (0.12)	0.03
Young-to-middle adulthood (20–54)	2883	410	0.0015 (0.05)	0.0014 (0.05)	0.0025 (0.08)	0.0031 (0.10)	0.03
Postmenopausal (55+)	2154	350	0.0019 (0.06)	0.0018 (0.06)	0.0033 (0.11)	0.004 (0.13)	0.03
Males							
Early childhood (1–5)	823	120	0.0037 (0.12)	0.0034 (0.11)	0.0067 (0.22)	0.0087 (0.29)	0.03
Middle childhood (6–9)	612	94	0.0034 (0.11)	0.0031 (0.10)	0.0063 (0.21)	0.0083 (0.28)	0.03
Adolescence (10–19)	1428	210	0.0027 (0.09)	0.0025 (0.08)	0.0053 (0.18)	0.0071 (0.24)	0.03
Young-to-middle adulthood (20–54)	2576	390	0.0025 (0.08)	0.0025 (0.08)	0.0043 (0.14)	0.0052 (0.17)	0.03
Late adulthood (55+)	2146	340	0.0023 (0.08)	0.0023 (0.08)	0.0038 (0.13)	0.0045 (0.15)	0.03

*ADI* acceptable daily intake, *NHANES* National Health and Nutrition Examination Survey, *JECFA* Joint Food and Agriculture Organization of the United Nations/World Health Organization Expert Committee on Food Additives.

^a^Usual daily intake is based on two non-consecutive 24-h recalls from NHANES dietary recall data (2015–2018).

^b^National Cancer Institute method for estimating the usual daily intake of episodically consumed foods (Tooze et al. [34]).

^c^The hazard quotient is defined as the ratio of the intake estimate to the ADI for melengestrol acetate established by JECFA, 0–0.03 µg/kg/day. A hazard quotient greater than 1 indicates that the melengestrol acetate intake estimate exceeded the established ADI. The estimated proportion of the population exceeding the ADI is 0.

^d^Total number of NHANES sample persons in demographic category who completed the NHANES dietary questionnaire.

^e^Number of NHANES sample persons consuming beef on both of the two dietary recall days. This set of individuals is used to estimate logistic and linear mixed models for predicting usual daily intake, with a sample size of 50 or greater recommended to ensure sufficient stability of the models. Subsequently, the models are used in Monte Carlo simulations to estimate usual daily intake for all individuals (see “Total n” column), including those not reporting beef consumption in the two 24-h recalls conducted by NHANES, using demographic and other data to predict long-term daily intake. This approach is described in greater detail in Tooze et al. [34].

^f^Exposure scenarios are defined by the assumed concentration of growth hormone in beef consumption reported by NHANES participants, as measured in purchased retail beef products using laboratory methods described elsewhere. “Typical” intake assumes the mean concentration was consumed; “high” intake assumes the 95th percentile was consumed; and “max” intake assumes the highest observed concentration was consumed.

**Table 3:** Estimates of usual daily progesterone intake from beef consumption, across various demographic groups and intake scenarios, in a nationally representative US sample from the National Health and Nutrition Examination Survey, 2015–2018.

Demographic category (age range in years)			Progesterone intake from estimated usual daily intake^ab^ of beef, µg/kg/day (Hazard quotient)^c^				
	Total *n*^d^	Eaters *n*^e^	Mean	Median	95th%	99th%	ADI (µg/kg/day)
Typical intake scenario^f^							
Females							
Early childhood (1–5)	798	120	0.0021 (<0.01)	0.0019 (<0.01)	0.0039 (<0.01)	0.005 (<0.01)	30
Middle childhood (6–9)	605	84	0.0015 (<0.01)	0.0014 (<0.01)	0.0028 (<0.01)	0.0038 (<0.01)	30
Adolescence (10–19)	1430	220	0.001 (<0.01)	0.00091 (<0.01)	0.002 (<0.01)	0.0027 (<0.01)	30
Young-to-middle adulthood (20–54)	2883	410	0.0012 (<0.01)	0.0011 (<0.01)	0.002 (<0.01)	0.0024 (<0.01)	30
Postmenopausal (55+)	2154	350	0.0015 (<0.01)	0.0014 (<0.01)	0.0025 (<0.01)	0.0031 (<0.01)	30
Males							
Early childhood (1–5)	823	120	0.0029 (<0.01)	0.0027 (<0.01)	0.0052 (<0.01)	0.0069 (<0.01)	30
Middle childhood (6–9)	612	94	0.0026 (<0.01)	0.0024 (<0.01)	0.0049 (<0.01)	0.0065 (<0.01)	30
Adolescence (10–19)	1428	210	0.0021 (<0.01)	0.0019 (<0.01)	0.0041 (<0.01)	0.0054 (<0.01)	30
Young-to-middle adulthood (20–54)	2576	390	0.002 (<0.01)	0.0019 (<0.01)	0.0033 (<0.01)	0.004 (<0.01)	30
Late adulthood (55+)	2146	340	0.0018 (<0.01)	0.0017 (<0.01)	0.0029 (<0.01)	0.0035 (<0.01)	30
High intake scenario^f^							
Females							
Early childhood (1–5)	798	120	0.011 (<0.01)	0.0097 (<0.01)	0.02 (<0.01)	0.026 (<0.01)	30
Middle childhood (6–9)	605	84	0.0076 (<0.01)	0.007 (<0.01)	0.014 (<0.01)	0.02 (<0.01)	30
Adolescence (10–19)	1430	220	0.0052 (<0.01)	0.0047 (<0.01)	0.01 (<0.01)	0.014 (<0.01)	30
Young-to-middle adulthood (20–54)	2883	410	0.006 (<0.01)	0.0058 (<0.01)	0.01 (<0.01)	0.012 (<0.01)	30
Postmenopausal (55+)	2154	350	0.0076 (<0.01)	0.0073 (<0.01)	0.013 (<0.01)	0.016 (<0.01)	30
Males							
Early childhood (1–5)	823	120	0.015 (<0.01)	0.014 (<0.01)	0.027 (<0.01)	0.035 (<0.01)	30
Middle childhood (6–9)	612	94	0.013 (<0.01)	0.012 (<0.01)	0.025 (<0.01)	0.033 (<0.01)	30
Adolescence (10–19)	1428	210	0.011 (<0.01)	0.0098 (<0.01)	0.021 (<0.01)	0.027 (<0.01)	30
Young-to-middle adulthood (20–54)	2576	390	0.01 (<0.01)	0.0097 (<0.01)	0.017 (<0.01)	0.021 (<0.01)	30
Late adulthood (55+)	2146	340	0.0092 (<0.01)	0.0089 (<0.01)	0.015 (<0.01)	0.018 (<0.01)	30
Max intake scenario^f^							
Females							
Early childhood (1–5)	798	120	0.03 (<0.01)	0.027 (<0.01)	0.056 (<0.01)	0.072 (<0.01)	30
Middle childhood (6–9)	605	84	0.021 (<0.01)	0.02 (<0.01)	0.04 (<0.01)	0.055 (<0.01)	30
Adolescence (10–19)	1430	220	0.015 (<0.01)	0.013 (<0.01)	0.029 (<0.01)	0.038 (<0.01)	30
Young-to-middle adulthood (20–54)	2883	410	0.017 (<0.01)	0.016 (<0.01)	0.028 (<0.01)	0.035 (<0.01)	30
Postmenopausal (55+)	2154	350	0.021 (<0.01)	0.02 (<0.01)	0.036 (<0.01)	0.045 (<0.01)	30
Males							
Early childhood (1–5)	823	120	0.041 (<0.01)	0.038 (<0.01)	0.075 (<0.01)	0.098 (<0.01)	30
Middle childhood (6–9)	612	94	0.038 (<0.01)	0.035 (<0.01)	0.07 (<0.01)	0.093 (<0.01)	30
Adolescence (10–19)	1428	210	0.03 (<0.01)	0.028 (<0.01)	0.059 (<0.01)	0.077 (<0.01)	30
Young-to-middle adulthood (20–54)	2576	390	0.028 (<0.01)	0.027 (<0.01)	0.048 (<0.01)	0.058 (<0.01)	30
Late adulthood (55+)	2146	340	0.026 (<0.01)	0.025 (<0.01)	0.042 (<0.01)	0.051 (<0.01)	30

*ADI* acceptable daily intake, *NHANES* National Health and Nutrition Examination Survey, *JECFA* Joint Food and Agriculture Organization of the United Nations/World Health Organization Expert Committee on Food Additives.

^a^Usual daily intake is based on two non-consecutive 24-h recalls from NHANES dietary recall data (2015–2018).

^b^National Cancer Institute method for estimating the usual daily intake of episodically consumed foods (Tooze et al. [34]).

^c^The hazard quotient is defined as the ratio of the intake estimate to the ADI for progesterone established by JECFA, 0–30 µg/kg/day. An HQ greater than 1 indicates that the progesterone intake estimate exceeded the established ADI. The estimated proportion of the population exceeding the ADI is 0.

^d^Total number of NHANES sample persons in demographic category who completed the NHANES dietary questionnaire.

^e^Number of NHANES sample persons consuming beef on both of the two dietary recall days. This set of individuals is used to estimate logistic and linear mixed models for predicting usual daily intake, with a sample size of 50 or greater recommended to ensure sufficient stability of the models. Subsequently, the models are used in Monte Carlo simulations to estimate usual daily intake for all individuals (see “Total n” column), including those not reporting beef consumption in the two 24-h recalls conducted by NHANES, using demographic and other data to predict long-term daily intake. This approach is described in greater detail in Tooze et al. [34].

^f^Exposure scenarios are defined by the assumed concentration of growth hormone in beef consumption reported by NHANES participants, as measured in purchased retail beef products using laboratory methods described elsewhere. “Typical” intake assumes the mean concentration was consumed; “high” intake assumes the 95th percentile was consumed; and “max” intake assumes the highest observed concentration was consumed.

Selected characteristics of the estimated distribution for usual daily T intake are provided in Supplementary Table S6. No estimated intakes exceeded the ADI of 2 µg/kg/day [37]. The highest estimate of usual daily T intake was for males in early childhood (ages 1–5), where the estimated 99^th^ percentile of intake under the max intake scenario was 0.0092 µg/kg/day, corresponding to an HQ of <0.01.

### Short-term daily intake using two-day averages

We calculated two-day average intake estimates (µg/kg/day) by averaging HGP intake over the two non-consecutive 24HRs, to estimate short-term (i.e., days to weeks) daily intake. Characteristics of the estimated intake distribution were higher under this approach compared to the NCI method, which was expected given the NCI method targets usual (i.e., “long-term”) intake. For MGA, under the max intake scenario, the 99^th^ percentile intake among females in early childhood (ages 1–5) was approximately equivalent to the ADI of 0.03 µg/kg/day [14]. Among boys in early childhood, the 99^th^ percentile was 0.036 µg/kg/day, exceeding the ADI and corresponding to an HQ of 1.19 (Supplementary Table S7). Under the typical intake scenario, estimated intake distributions were very low compared to the ADI, with the highest median intake for females in early childhood (ages 1–5) with 0.00021 µg/kg/day, corresponding to an HQ of 0.01. Two-day average intake estimates for P and T were also very low compared to the ADIs (Supplementary Tables S8–S9).

### Estimated MGA intake by race/ethnicity and income

**Fig. 1:**
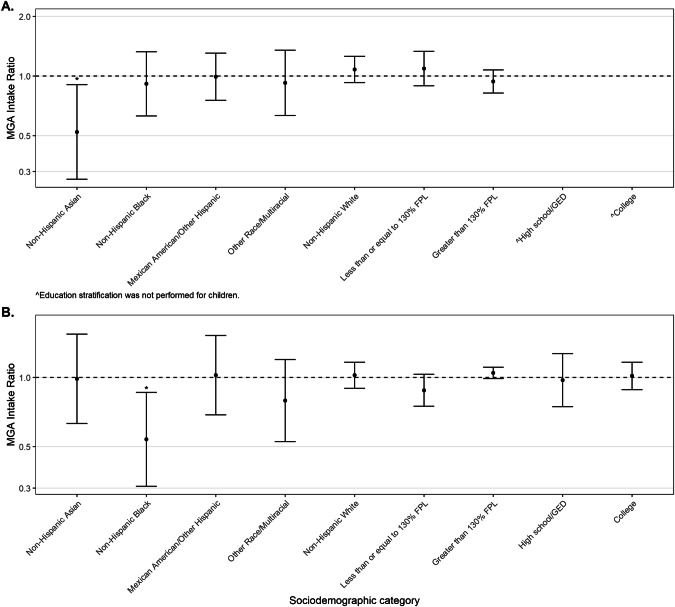
Ratios and 95% confidence intervals of estimated median usual daily MGA intake, comparing various sociodemographic groups to overall intake in NHANES (2015–18). **A** Estimated median usual daily MGA intake by children (1–19 years). **B** Estimated median usual daily MGA intake by women (20+ years). *p < 0.05 comparing the subgroup and overall intake. ^Education stratification was not performed for children. FPL federal poverty line, GED General Education Development test (equivalent of high school graduate), MGA melengestrol acetate, NHANES National Health and Nutrition Examination Survey.

For children (ages 1–19) and women (ages 20+), we estimated ratios of median usual daily MGA intake (intake ratios, IR) among subgroups defined by race/ethnicity, poverty, and education, to the overall population of children or women (Fig. 1). This was done under the typical intake scenario to maximize representativeness of the results to the US population. Among children, non-Hispanic Asians had about half the MGA intake of all children (IR = 0.52, 95% CI: 0.30, 0.91; *p* = 0.02). Non-Hispanic Black women had approximately half the estimated MGA intake compared to all women (IR = 0.54, 95% CI: 0.34, 0.86; *p* = 0.01). Estimated median intake of MGA was numerically higher among women in households with an annual income >130% of the FPL, compared to all women, though this difference was not statistically significant (IR = 1.05, 95% CI: 0.99, 1.11; *p* = 0.11). There was no significant difference in estimated MGA intake by education level among women.

## Discussion

We measured HGP concentrations in nearly 400 samples of retail beef/fat to estimate distributions of long-term (i.e., usual) and short-term (i.e., over several days to weeks) daily intake (µg HGP/kg bodyweight/day) of MGA, P, and T, from beef consumed by among US children and adults. We then compared estimates to established ADI limits. Under the typical intake scenario, which aims to represent intake for the average US individual, estimated long-term daily intakes for all HGP were well-below their respective ADIs. Among young boys ages 1–5, the estimated 99^th^ percentile of long-term daily MGA intake under the max intake scenario, which aims to establish an upper bound of intake, corresponded to 29% of the ADI. In contrast, estimates for short-term daily intake equaled (for young girls, ages 1–5) or exceeded (for young boys, ages 1–5) the ADI for MGA. Non-Hispanic Black women and non-Hispanic Asian children had lower estimated MGA intake than the overall populations of women and children, respectively.

Others have previously reported concentrations of various HGP including P, EpiT, T, and estradiol in retail beef [16, 17, 19–21], as well as in control and treated animals in experimental settings [10, 18]. In one study reporting MGA residues in beef tissues, Daxenberger et al. treated heifers with a maximum MGA dose of 5 mg/day, and subsequently recorded a maximum concentration of MGA in muscle of 1.3 pg/mg, whereas the highest concentration we recorded in a non-fat sample was in liver with 2.38 pg/mg [4]. Notably, Daxenberger et al. also recorded a mean concentration of 59 pg/mg in perirenal fat from the same animals, far exceeding the current maximum residue level for MGA of 18 pg/mg [14]. The highest concentration of MGA that we recorded was 4.07 pg/mg, in an adipose subsample trimmed from a ribeye steak. Had we used the mean concentration in perirenal fat reported by Daxenberger (59 pg/mg) to establish an even more conservative upper bound for potential human intake, our estimates of usual daily MGA intake would be higher, but still low relative to the ADI, given that fat constitutes a relatively small portion (~15%) of consumed beef on a weight basis. Also, MGA levels may differ in perirenal *versus* muscle-adjacent adipose, and thus perirenal concentrations may be inappropriate for intake assessment. In a separate study of 671 heifer carcasses in Canada, levels of MGA in fat never exceeded 30 pg/mg [43]. These levels and their elevation in fat are in keeping with those from the earlier tissue distribution analyses performed by Krzeminski and colleagues [44].

We found that short-term MGA intake via consumed beef products may exceed the ADI in some young children. Feeding MGA to feedlot heifers suppresses their ovarian cyclicity, thereby increasing their liveweight gain, where MGA acts via a progestin-like mechanism [45]. The WHO developed the ADI for MGA based on a single study of 8 adult female cynomolgus monkeys treated with MGA at three dose levels over a period of about 100 days (two animals per dose group, including a control) [25], with endpoints measured including hormone concentrations (e.g., LH, estradiol, P), ovulation, and normal menstruation. Because the study described biological effects at all dose levels, the WHO applied a safety factor of 200 to the lowest dose group to account for potential effects at even lower levels, as well as to account for interspecies and intraspecies differences. However, several aspects of this study suggest that the resulting ADI may not be sufficiently health-protective, particularly for children. Exposure to HGP that alters menstrual cyclicity, such as MGA, may alter endocrine function and pubertal timing in children at lower doses than for adults [9, 46]. In fact, few toxicological studies of MGA and other HGP have considered potentially-sensitive and detrimental developmental windows of exposure, such as in the prenatal or early postnatal periods [9]. Furthermore, while our short-term intake estimates, which represent intake over the course of a few days or weeks, are not strictly comparable to the chronic intake implied by the concept of an ADI, the monkey study’s dosing period of only 100 days, or three menstrual cycles, is also likely insufficient to evaluate the effects of truly long-term or daily intake. Additionally, the study sample size was very limited, with only two animals per dose group, likely precluding the detection of smaller, but nonetheless consequential, effects on reproductive function, particularly those that may result from chronic exposures [47]. That study also did not identify a no-effect level, and there is no guarantee that the safety factor can sufficiently account for this gap in evidence. Overall, our results suggest that short-term MGA intake levels in US children are high enough to warrant additional toxicological study of this HGP to fill several gaps left by the current ADI [9, 48].

Given that progestins that form part of hormone replacement therapy can increase breast cancer risk in women [47], there is some concern that MGA may promote carcinogenesis [47]. In fact, some studies have shown adverse effects of MGA on mammary development or carcinogenesis in experimental models, although those experiments involved administering high levels of MGA [25]. Whether there are any underlying links between the intake of residual low levels of MGA, either as the parent compound or its less potent metabolites, and cancer, remains to be explored [49]. In particular, there is a lack of low-dose, lifetime oral toxicity studies in the published literature, which would better reflect the chronic nature of beef intake, and the route of exposure (*versus* subcutaneous injection) which can affect the bioavailability of MGA and its subsequent effects.

The most commonly used HGP in the United States is Revalor®, a mixture of EB and TBA, which is administered to approximately 72% of all feedlot cattle [1]. Notably, we did not detect EB in any sample, and only detected TBA in one sample of fat trimmed from a ribeye steak. By contrast, an analysis in France detected low-level residues of EB in the liver, kidney, and muscle of cattle implanted with E2 and TBA [16]. Our findings suggest that these hormones are being used in the US according to recommendations. Consistent with our findings, studies in Austria and Taiwan did not find concentrations of Z above quantitation limits [17, 50]. The mean concentration of P we measured in fat (4.9 pg/mg) was lower than that found in fat tissue of male calf controls (5.8 pg/mg) and those treated with Implix-BM (12.5 pg/mg), a product releasing E2 and P, in a prior study [51]. However, even if we had used these higher concentrations for our intake assessment, the ADI would not have been exceeded. Similar to studies in other countries [16, 52], our intake estimates for T from beef were relatively low.

Endogenous hormones like P and T are also present in dairy products. For example, Hartmann et al. [53] found mean concentrations of P in butter (141 pg/mg), yogurt (13.3 pg/mg), and milk (9.81 pg/mg) that were higher than the mean concentration we found in beef fat (4.91 pg/mg) and non-fat tissues (1.84 pg/mg). Courant et al. reported mean concentrations of EpiT and T in retail milk products such that consuming a serving of whole milk (~8 fl. oz.) would deliver a two- to three-fold higher intake of these compounds than a serving of fat-trimmed beef (~3 oz.) [16]. When considering total daily intake of EpiT, T, and P from all dietary sources, our estimates of intake from consumed beef should be viewed as minimum total intakes, likely underrepresenting total intake for most people given these other potential dietary sources.

Non-Hispanic Black women had lower estimated MGA intake than other women, while non-Hispanic Asian children had a lower estimated MGA intake than other children. Additionally, there was weak evidence that women from higher income households had higher estimated MGA intake than other women, though this difference was not statistically significant. These differences in intake are attributable to differences between these groups in (1) the amount of beef consumed relative to bodyweight, and (2) the proportion of consumed beef comprised of fat, which corresponds to higher intake of HGP given HGP concentrations are higher in fat. Because intake estimates for all HGP were based exclusively on the consumption of beef, which was estimated by the same procedure for each HGP, socioeconomic differences in estimated MGA intake would extend to all HGP. In the early 2000’s, the USDA reported that per capita beef consumption was higher among lower income families compared to higher income families and higher among Black individuals compared to White individuals [54]. In contrast, we found higher HGP intake among higher income families than lower income families, and lower intake among Black women compared to others. This difference may be attributable to what the USDA qualified as beef consumption (e.g., the inclusion of the beef ingredients of complex foods, compared to counting beef-only foods), our accounting for potential group differences in the fat content of beef consumed, and our calculation of intake on a per-bodyweight basis rather than per capita. Interestingly, race/ethnicity- and income-based differences for MGA intake were not consistent across women and children, suggesting that differences in beef intake may shift over the life course due to relative changes in dietary patterns, types of beef products consumed, and bodyweight. In turn, differences in HGP intake may alter the incidence and severity of hormone-sensitive adverse health outcomes, potentially exacerbating health disparities.

The present study has some notable limitations. First, we obtained a non-random sample of retail cuts of beef in California, which may not reflect beef consumed across the US. However, we made efforts to be as inclusive and representative as possible within our sampling frame. We analyzed hundreds of retail beef products purchased from a wide range of retailers, including national chains and local ethnic markets, in both major urban centers and agricultural areas. Second, we did not detect HGP in a majority of samples, resulting in intake estimates that were mostly based on non-detects that were uniformly imputed as $\frac{{LOD}}{\sqrt{2}}$. However, usual daily intakes of MGA, P, and T, even under the max intake scenario, were generally very low relative to their ADI. Therefore, our findings with respect to the current ADIs would likely not change with even more sensitive detection of HGP. Third, several assumptions were made in estimating beef intake using NHANES data. For example, we assumed all beef fat was retained during cooking and was consumed which, if false for most people, would contribute to overestimation of HGP intake. However, usual daily intakes of each HGP were still very low relative to their respective ADIs, and thus our conclusions are unlikely to be sensitive to this assumption. Fourth, we were unable to account for potential differences in HGP concentrations by cut because (1) there was insufficient resolution in the dietary recall information to distinguish different cuts consumed, and (2) among our retail samples, sample size by cut was insufficient to detect statistically significant differences. Even if we utilized the observed non-significant differences between concentrations by cut, they were too small in magnitude to be consequential for the analysis, particularly compared to the differences between subsampled fat and all other samples.

Because the measurement error structure of the NHANES 24HR as a measure of beef intake is unknown, it is possible that both the estimated short-term and usual intake distributions are biased. Because the NCI method accounts for within-person random error [34], bias in estimated usual intake distributions is the net effect of any systematic biases left untouched by the modeling approach. Using the model framework outlined by Kipnis et al. [55] the net effect of systematic bias can be characterized by 1) a group-level linear relationship between the expected value of 24HR intake and true usual intake (i.e. “intake-related bias”) and 2) the variance of individual-level deviations from the group-level relationship (i.e. “person-specific bias”). If the group-level trend is towards over-reporting of usual intake, then both components lead to over-estimation of the upper percentiles (i.e., the tails of the estimated distributions are longer than the true distribution). On the other hand, if the group-level trend is towards under-reporting, the two components work at cross purposes and depending on their respective magnitudes, could result in under- or over-estimation of the upper percentiles. The effect of random within-person error that is reduced, but not eliminated, in the short-term distributions is a tendency to over-estimate the upper percentiles of those distributions. Our results are conservative if both systematic components exaggerate the upper percentiles. Of more concern is the mixed-case scenario where a severe group level trend for underreporting (that biases the upper percentiles downward) overwhelms the exaggerating effects of person-specific bias. The highest estimated HQs for MGA at the 99^th^ percentile in the max intake scenario were approximately 0.33. If the true 99^th^ percentile of beef intake was three times the estimate (relative underestimation by 67%) due to the combination of systematic bias components, the true HQs would equal or exceed 1, in contrast to our estimates. Evidence from validation studies of 24HRs using recovery biomarkers is scant, and practically nonexistent for children. However, Kipnis et al. [55] and more recently, Kirkpatrick et al. [56] found that 24HR-based assessment of protein intake (albeit in adults) was substantially better than, for example, assessment of energy intake. In particular, Kirkpatrick et al. found that even after adjusting for within-person variation in 24HR protein, the net effect of systematic error is modest attenuation of relative risks, which would suggest that upper percentiles would be, if anything, overestimated by the NCI Method [56].

After careful consideration of the limitations outlined above, we conclude that usual daily intakes of P, T, and MGA from beef consumed by US children and adults is likely well-below the respective ADIs of these HGP, with significant margins of safety. We found that short-term intake of MGA among young boys ages 1–5 under the max intake scenario may exceed the ADI, although the potential health consequences of short-term, high-level MGA intake in children are unknown. While the present work suggests current levels of beef-based HGP intake are not of public health concern, a more comprehensive evaluation will require toxicologic studies of long-term, low-dose exposure to HGP, especially during sensitive developmental windows, which do not currently exist [7].

## Supplementary information

Supplementary Material

Supplementary Material Table S1

## Data Availability

Dietary recall, demographic, and income data from the National Health and Nutrition Examination Survey (NHANES) are publicly available online, for the 2015–2016 (https://wwwn.cdc.gov/nchs/nhanes/continuousnhanes/overview.aspx?BeginYear=2015) and 2017–2018 (https://wwwn.cdc.gov/nchs/nhanes/continuousnhanes/overview.aspx?BeginYear=2017) cycles. Data on hormone levels in beef are not available.

## References

[1] APHIS. The use of growth-promoting implants in US feedlots. U.S. Department of Agriculture, Animal and Plant Health Inspection Service, Veterinary Services, Centers for Epidemiology and Animal Health. 2013. https://www.govinfo.gov/app/details/GOVPUB-A101-PURL-gpo83125

[2] Fossler C. NAHMS 2021 Feedlot study. American Association of Bovine Practitioners Conference. 2023;19–20 10.21423/aabppro20228589.

[3] Animal Drugs @ FDA. 2023. Available from: https://animaldrugsatfda.fda.gov/adafda/views/#/search.

[4] Daxenberger A, Meyer K, Hageleit M, Meyer HHD. Detection of melengestrol acetate residues in plasma and edible tissues of heifers. Vet Q. 1999;21:154–8. 10568006 10.1080/01652176.1999.9695011

[5] Yager JD, Davidson NE. Estrogen Carcinogenesis in Breast Cancer. N Engl J Med. 2006;354:270–82. 16421368 10.1056/NEJMra050776

[6] Xu S, Murtagh S, Han Y, Wan F, Toriola AT. Breast Cancer Incidence Among US Women Aged 20 to 49 Years by Race, Stage, and Hormone Receptor Status. JAMA Netw Open. 2024;7:e2353331. 38277147 10.1001/jamanetworkopen.2023.53331 PMC10818222

[7] Vandenberg LN, Colborn T, Hayes TB, Heindel JJ, Jacobs DR Jr, et al. Hormones and Endocrine-Disrupting Chemicals: Low-Dose Effects and Nonmonotonic Dose Responses. Endocr Rev. 2012;33:378–455. 22419778 10.1210/er.2011-1050 PMC3365860

[8] Lee JE, Jung HW, Lee YJ, Lee YA. Early-life exposure to endocrine-disrupting chemicals and pubertal development in girls. Ann Pediatr Endocrinol Metab. 2019;24:78–91. 31261471 10.6065/apem.2019.24.2.78 PMC6603611

[9] Nachman KE, Smith TJS. Hormone Use in Food Animal Production: Assessing Potential Dietary Exposures and Breast Cancer Risk. Curr Environ Health Rep. 2015;2:1–14. 26231238 10.1007/s40572-014-0042-8

[10] Stephany RW. Hormonal growth promoting agents in food producing animals. Handb Exp Pharmacol. 2010;195:355–67. 10.1007/978-3-540-79088-4_16 20020373

[11] Lau CS, Fulgoni VL, Van Elswyk ME, McNeill SH. Trends in Beef Intake in the United States: Analysis of the National Health and Nutrition Examination Survey, 2001-2018. Nutrients. 2023;15:2475. 37764720 10.3390/nu15183936 PMC10537678

[12] Andersson A, Skakkebaek N. Exposure to exogenous estrogens in food: possible impact on human development and health. Eur J Endocrinol. 1999;140:477–85. 10366402 10.1530/eje.0.1400477

[13] Handa Y, Fujita H, Honma S, Minakami H, Kishi R. Estrogen concentrations in beef and human hormone-dependent cancers. Ann Oncol. 2009;20:1610–1. 19628569 10.1093/annonc/mdp381

[14] WHO | JECFA. 2023. Available from: https://apps.who.int/food-additives-contaminants-jecfa-database/Home/Chemical/5125.

[15] Food Safety Commission of Japan. Melengestrol Acetate (Veterinary Medicinal Products). Food Saf. 2017;5:164–8. 10.14252/foodsafetyfscj.2017009s PMC6989191 32231940

[16] Courant F, Antignac JP, Laille J, Monteau F, Andre F, Le Bizec B. Exposure Assessment of Prepubertal Children to Steroid Endocrine Disruptors. 2. Determination of Steroid Hormones in Milk, Egg, and Meat Samples. J Agric Food Chem. 2008;56:3176–84. 18412364 10.1021/jf800096f

[17] Hsieh MK, Chen H, Chang JL, She WS, Chou CC. Electrochemical Detection of Zeranol and Zearalenone Metabolic Analogs in Meats and Grains by Screen-Plated Carbon-Plated Disposable Electrodes. FNS. 2013;04:31–8.

[18] Kleinova M, Zöllner P, Kahlbacher H, Hochsteiner W, Lindner W. Metabolic profiles of the mycotoxin zearalenone and of the growth promoter zeranol in urine, liver, and muscle of heifers. J Agric Food Chem. 2002;50:4769–76. 12166958 10.1021/jf020160p

[19] López-García M, Romero-González R, Garrido Frenich A. Determination of steroid hormones and their metabolite in several types of meat samples by ultra high performance liquid chromatography—Orbitrap high resolution mass spectrometry. J Chromatogr A. 2018;1540:21–30. 29397061 10.1016/j.chroma.2018.01.052

[20] Chafi S, Ballesteros E. A Simple, Efficient, Eco-Friendly Sample Preparation Procedure for the Simultaneous Determination of Hormones in Meat and Fish Products by Gas Chromatography—Mass Spectrometry. Foods. 2022;11:3095. 36230170 10.3390/foods11193095 PMC9562678

[21] Nazli B, Çolak H, Aydin A, Hampikyan H. The Presence of Some Anabolic Residues in Meat and Meat Products Sold in Ýstanbul. Turkish J Vet Anim Sci. 2005;29:691–9.

[22] Food Safety and Inspection Service. Report on the Food Safety and Inspection Service’s Microbiological and Residue Sampling Programs. United States Department of Agriculture; 2011. Available from: https://www.fsis.usda.gov/sites/default/files/media_file/2021-02/FSIS_Sampling_Programs_Report.pdf.

[23] UNITED STATES National Residue Program for Meat, Poultry, and Egg Products: FY 2019 RESIDUE SAMPLE RESULTS. Office of Public Health Science, Food Safety and Inspection Service, United States Department of Agriculture; 2019. Available from: https://www.fsis.usda.gov/sites/default/files/media_file/2020-07/fy2019-red-book.pdf.

[24] Food Safety and Inspection Service Annual Sampling Plan: Fiscal Year 2024. Food Safety and Inspection Service, U.S. Department of Agriculture; 2024. Available from: https://www.fsis.usda.gov/sites/default/files/media_file/documents/FSIS-Annual-Sampling-Plan-FY2024.pdf.

[25] Food and Agriculture Organization of the United Nations, World Health Organization. Evaluation of Certain Veterinary Drug Residues in Food: Seventieth Report of the Joint FAO/WHO Expert Committee on Food Additives. Geneva, Switzerland; 2008. p. 46–56. (WHO Technical Report Series). Report No.: 62nd. Available from: https://apps.who.int/iris/bitstream/handle/10665/44085/WHO_TRS_954_eng.pdf?sequence=1.

[26] Centers for Disease Control and Prevention (CDC). National Center for Health Statistics (NCHS). National Health and Nutrition Examination Survey Data 2015-2016. U.S. Department of Health and Human Services, Centers for Disease Control and Prevention; 2018. Available from: https://wwwn.cdc.gov/nchs/nhanes/search/datapage.aspx?Component=Dietary&Cycle=2015-2016.

[27] Centers for Disease Control and Prevention (CDC). National Center for Health Statistics (NCHS). National Health and Nutrition Examination Survey Data 2017-2018. U.S. Department of Health and Human Services, Centers for Disease Control and Prevention; 2020. Available from: https://wwwn.cdc.gov/nchs/nhanes/search/datapage.aspx?Component=Dietary&CycleBeginYear=2017.

[28] Agarwal S, Fulgoni VL. Contribution of Beef to Key Nutrient Intakes and Nutrient Adequacy in Pregnant and Lactating Women: NHANES 2011-2018 Analysis. Nutrients. 2024;16:981. 38613015 10.3390/nu16070981 PMC11013741

[29] FNDDS DOWNLOAD DATABASES: USDA ARS. 2023. Available from https://www.ars.usda.gov/northeast-area/beltsville-md-bhnrc/beltsville-human-nutrition-research-center/food-surveys-research-group/docs/fndds-download-databases/.

[30] Bowman SA, Clemens JC, Shimizu M, Friday JE, Moshfegh AJ. Food Patterns Equivalents Database 2015-2016: Methodology and User Guide [Online]. Food Surveys Research Group, Beltsville Human Nutrition Research Center, Agricultural Research Service, U.S. Department of Agriculture, Beltsville, Maryland. 2018. Available at: https://www.ars.usda.gov/fsrg.

[31] Bowman SA, Clemens JC, Friday JE, Moshfegh AJ. Food Patterns Equivalents Database 2017-2018: Methodology and User Guide [Online]. Food Surveys Research Group, Beltsville Human Nutrition Research Center, Agricultural Research Service, U.S. Department of Agriculture, Beltsville, Maryland. 2020. Available at: http://www.ars.usda.gov/nea/bhnrc/fsrg.

[32] Mosburg M, Li Y, Helmes E, Falt TD, Trott JF, Solomon G, Hovey RC, Moeller BC. Determination of Hormonal Growth Promotants in Beef using Liquid Chromatography Tandem Mass Spectrometry. Drug Testing and Analysis. 2024; In Press. 10.1002/dta.3827 PMC12319502 39561982

[33] Hornung RW, Reed LD. Estimation of Average Concentration in the Presence of Nondetectable Values. Appl Occup Environ Hyg. 1990;5:46–51.

[34] Tooze JA, Midthune D, Dodd KW, Freedman LS, Krebs-Smith SM, Subar AF, et al. A new statistical method for estimating the usual intake of episodically consumed foods with application to their distribution. J Am Diet Assoc. 2006;106:1575–87. 17000190 10.1016/j.jada.2006.07.003 PMC2517157

[35] Luo H, Dodd KW, Arnold CD, Engle-Stone R. Advanced dietary analysis and modeling: a deep dive into the National Cancer Institute method. J Nutr. 2022;152:2615–25. 36774127 10.1093/jn/nxac144 PMC9644173

[36] Chen TC, Clark J, Riddles MK, Mohadjer LK, Fakhouri THI. National Health and Nutrition Examination Survey, 2015-2018: Sample Design and Estimation Procedures. Vital- Health Stat. 2020;2:1–35. 33663649

[37] WHO | JECFA. 2023. Available from: https://apps.who.int/food-additives-contaminants-jecfa-database/Home/Chemical/2452.

[38] WHO | JECFA. 2023. Available from: https://apps.who.int/food-additives-contaminants-jecfa-database/Home/Chemical/3580.

[39] Citron K, Murthy DB, Shah B. Endocrine-Disrupting Chemicals in Children. Pediatrics Rev. 2024;45:111–5. 10.1542/pir.2022-005725 38296777

[40] Rattan S, Zhou C, Chiang C, Mahalingam S, Brehm E, Flaws JA. Exposure to endocrine disruptors during adulthood: Consequences for female fertility. J Endocrinol 2017;233:R109–29. 28356401 10.1530/JOE-17-0023 PMC5479690

[41] National Center for Health Statistics (U.S.), editor. Estimating usual dietary intake from National Health and Nutrition Examination Survey data using the National Cancer Institute method. Hyattsville, Maryland: U.S. Department of Health and Human Services, Centers for Disease Control and Prevention, National Center for Health Statistics; 2018. 54 p. (Vital and health statistics. Series 2, data evaluation and methods research). 29775432

[42] R Core Team. R: A Language and Environment for Statistical Computing. Vienna, Austria: R Foundation for Statistical Computing; 2023. Available from: https://www.R-project.org/.

[43] Neidert EE, Gedir RG, Milward LJ, Salisbury CD, Gurprasad NP, Saschenbrecker PW. Determination and qualitative confirmation of melengestrol acetate residues in beef fat by electron capture gas chromatography and gas chromatographic/chemical ionization mass spectrometry. J Agric Food Chem. 1990;38:979–81.

[44] Krzeminski LF, Cox BL, Gosline RE. Fate of radioactive melengestrol acetate in the bovine. J Agric Food Chem. 1981;29:387–91. 7229218 10.1021/jf00104a041

[45] Perry GA, Welshons WV, Bott RC, Smith MF. Basis of melengestrol acetate action as a progestin. Domest Anim Endocrinol. 2005;28:147–61. 15713363 10.1016/j.domaniend.2004.07.002

[46] Miller M, Marty M, Arcus A, Brown J, Morry D, Sandy M. Differences Between Children and Adults: Implications for Risk Assessment at California EPA. Int J Toxicol. 2002;21:403–18. 10.1080/10915810290096630 12396687

[47] Beral V, Million Women Study Collaborators. Breast cancer and hormone-replacement therapy in the Million Women Study. Lancet. 2003;362:419–27. 12927427 10.1016/s0140-6736(03)14065-2

[48] Kolok AS, Ali JM, Rogan EG, Bartelt-Hunt SL. The Fate of Synthetic and Endogenous Hormones Used in the US Beef and Dairy Industries and the Potential for Human Exposure. Curr Environ Health Rep. 2018;5:225–32. 29754262 10.1007/s40572-018-0197-9

[49] Jeong SH, Kang D, Lim MW, Kang CS, Sung HJ. Risk Assessment of Growth Hormones and Antimicrobial Residues in Meat. Toxicol Res. 2010;26:301–13. 24278538 10.5487/TR.2010.26.4.301 PMC3834504

[50] Metabolic Profiles of the Mycotoxin Zearalenone and of the Growth Promoter Zeranol in Urine, Liver, and Muscle of Heifers. 2023. Available from: https://pubs-acs-org.libproxy.berkeley.edu/doi/epdf/10.1021/jf020160p. 10.1021/jf020160p 12166958

[51] Hoffmann B, Evers P. Anabolic agents with sex hormone-like activities: problems of residues. In Rico AG (Ed.), Drug Residues Animals. Academic Press; 1986. pp 111–146.

[52] Fritsche S, Rumsey TS, Meyer HHD, Schmidt G, Steinhart H. Profiles of steroid hormones in beef from steers implanted with Synovex-S (estradiol benzoate and progesterone) in comparison to control steers. Z Lebensm Unters Forsch. 1999;208:328–31.

[53] Hartmann S, Lacorn M, Steinhart H. Natural occurrence of steroid hormones in food. Food Chem. 1998;62:7–20.

[54] Davis CG, Lin BH. Factors Affecting U.S. Beef Consumption. United States Department of Agriculture. 2005. Available from: https://www.ers.usda.gov/webdocs/outlooks/37388/29633_ldpm13502_002.pdf?v=8587.5.

[55] Kipnis V, Subar AF, Midthune D, Freedman LS, Ballard-Barbash R, Troiano RP, et al. Structure of dietary measurement error: results of the OPEN biomarker study. Am J Epidemiol. 2003;158:14–21. 12835281 10.1093/aje/kwg091

[56] Kirkpatrick SI, Troiano RP, Barrett B, Cunningham C, Subar AF, Park Y, et al. Measurement Error Affecting Web- and Paper-Based Dietary Assessment Instruments: Insights From the Multi-Cohort Eating and Activity Study for Understanding Reporting Error. Am J Epidemiol. 2022;191:1125–39. 35136928 10.1093/aje/kwac026 PMC9393065

