# Supplementary material for: Estimated human intake of endogenous and exogenous hormones from beef in the United States
Source: J Expo Sci Environ Epidemiol. 2024 Nov 7;35(3):362–74. doi: 10.1038/s41370-024-00727-1 (PMC12069096; doi:10.1038/s41370-024-00727-1)
Supplement: Supplementary file 1 — Supplementary Material [file 41370_2024_727_MOESM1_ESM.pdf]

Supplementary Material

Estimated Human Intake of Endogenous and Exogenous Hormones from Beef in the United States

Ruwan Thilakaratne[1], Rosemary Castorina[2], Gina Solomon[3], Mary M. Mosburg[4], Benjamin C. Moeller[4,5], Josephine F. Trott[6], Tara D. Falt[6], Ariadne Villegas Gomez[7], Kevin W. Dodd[8], Catherine Thomsen[9], Paul English[7], Xiang Yang[6], Annika Khan[2], Asa Bradman[10] & Russell C. Hovey[5,*]

[1]Division of Epidemiology, School of Public Health, University of California, Berkeley, Berkeley, CA, USA

[2]Center for Environmental Research and Community Health, School of Public Health, University of California, Berkeley, Berkeley, CA, USA

[3]Division of Occupational, Environmental, and Climate Medicine, University of California San Francisco, San Francisco, CA, USA

[4]Kenneth L. Maddy Equine Analytical Chemistry Laboratory, School of Veterinary Medicine, University of California, Davis, Davis, California, USA.

[5]Department of Molecular Biosciences, School of Veterinary Medicine, University of California, Davis, Davis, California, USA.

[6]Department of Animal Science, University of California, Davis, One Shields Ave, Davis, CA, USA

[7]Tracking California, Public Health Institute, Oakland, CA, USA

[8]Biometry Research Group, Division of Cancer Prevention, National Cancer Institute, Bethesda, MD, USA

[9]Zero Breast Cancer, San Rafael, CA, USA

[10]Department of Public Health, University of California, Merced, Merced, CA, USA

***Corresponding author:**

Russell C. Hovey, PhD

Department of Animal Science, University of California, Davis

One Shields Avenue, Davis, CA 95616-8521

E-mail: rchovey@ucdavis.edu

Table of Contents

Table S1. Foods containing beef, their beef ingredients, and corresponding numeric identifiers, from the United States Department of Agriculture Food and Nutrient Database for Dietary Studies, 2015-2018. Codes were used to identify beef-containing foods and estimate beef intake from dietary recall data in the 2015-2018 National Health and Nutrition Examination Survey. ... 4

Table S2. Acceptable daily intakes and health endpoints established by WHO/JECFA for five hormonal growth promotants detected in retail beef samples purchased for this study. 5

Table S3. Store locations by region and city where retail beef samples (N=321) were purchased between May 15, 2020 and May 9, 2022, to be analyzed for hormonal growth promotant concentrations. 6

Table S4. Types of retail beef/fat samples (N=397) analyzed for hormonal growth promotant concentrations. 7

Table S5. Product labeling of retail beef samples (N=321) analyzed for hormonal growth promotant concentrations. 8

Table S6. Detection rates and concentration distributions for each of five hormones, by fat tissue and non-fat (muscle, organs, ground, etc.) tissue. Samples below the limit of detection were imputed as the limit of detection divided by the square-root of 2, prior to calculation of distribution statistics. 9

Table S7. Estimates of usual daily testosterone intake from beef consumption, across various demographic groups and intake scenarios, in a nationally representative U.S. sample from the National Health and Nutrition Examination Survey, 2015-2018.. 10

Table S8. Two-day average estimates of daily melengestrol acetate intake from beef consumption, across various demographic groups and intake scenarios, in the U.S. population (NHANES 2015-2018). 15

Table S9. Two-day average estimates of daily progesterone intake from beef consumption, across various demographic groups and intake scenarios, in the U.S. population (NHANES 2015-2018)... 19

Table S10. Two-day average estimates of daily total testosterone intake (sum of testosterone and epitestosterone) from beef consumption, across various demographic groups and intake scenarios, in the U.S. population (NHANES 2015-2018). 23

Table S1. Foods containing beef, their beef ingredients, and corresponding numeric identifiers, from the United States Department of Agriculture Food and Nutrient Database for Dietary Studies, 2015-2018. Codes were used to identify beef-containing foods and estimate beef intake from dietary recall data in the 2015-2018 National Health and Nutrition Examination Survey.

See associated Excel spreadsheet supplementary file, "Table S1.xlsx".

Table S2. Acceptable daily intakes and health endpoints established by WHO/JECFA for five hormonal growth promotants detected in retail beef samples purchased for this study.

HGP	ADI (µg/kg bw/day)	Health endpoint
Epitestosterone	None[a]	–
Melengestrol Acetate	0-0.03	Menstrual cycle effects (reduced menstruation and ovulation; increased incidence of changed cycles)[b]
Progesterone	0-30	Effects on uterus (increased withdrawal bleeding)[c]
Testosterone	0-2	Restoration normal sexual desire, erection, ejaculation, and general well-being in eunuchs[d]

ADI: acceptable daily intake; HGP: hormonal growth promotant; LOEL: lowest observed effect level; NOEL: no observed effect level; WHO/JECFA: World Health Organization/United Nations Joint Expert Committee on Food Additives

[a]No ADI has been established for epitestosterone.

[b]Description of the pivotal study is a direct quote from the WHO website for this HGP: https://apps.who.int/food-additives-contaminants-jecfa-database/Home/Chemical/5125

[c]Description of the pivotal study is a direct quote from the WHO website for this HGP: https://apps.who.int/food-additives-contaminants-jecfa-database/Home/Chemical/2875

[d]Description of the pivotal study is a direct quote from the WHO website for this HGP: https://apps.who.int/food-additives-contaminants-jecfa-database/Home/Chemical/2452

Table S3. Store locations by region and city where retail beef samples (N=321) were purchased between May 15, 2020 and May 9, 2022, to be analyzed for hormonal growth promotant concentrations.

Location	Percent (N=321)
Bay Area	
San Francisco	5.9
Berkeley	4.0
Oakland	3.1
Central Valley	
Fresno	6.5
Clovis	1.6
Salinas	33.0
Southern California	
Anaheim	10.9
Los Angeles	7.5
Rowland Heights	5.0
La Puente	5.0
Eastvale	3.7
Fullerton	3.7
Orange	3.7
Corona	2.5
Santa Ana	1.9
City of Industry	1.9

Table S4. Types of retail beef/fat samples (N=397) analyzed for hormonal growth promotant concentrations.

Product Category	Percent (N=397)
Fat[a]	19.1
Chuck	15.1
Ground beef, 11-20 % fat	10.3
Ground beef, 0-10 % fat	10.1
Round	6.8
Liver	6.0
Stomach	6.0
Loin	5.8
Rib	5.8
Ground beef, 21-30 % fat	4.0
Multiple cuts	4.0
Ground beef, unknown % fat	3.0
Subprimal	1.8
Heart	1.3
Tongue	0.5
Tail	0.3

[a]Fat samples consist of trimmings from purchased retail cuts, not pure beef fat products.

Table S5. Product labeling of retail beef samples (N=321) analyzed for hormonal growth promotant concentrations.

Label	Percent (N=321)
Hormone Free	
Yes	9.7
Not declared	90.3
Organic	
Yes	5.3
Not declared	94.7
USDA/Uruguay Inspection Seal	
Yes	20.9
Wrapped by butcher	59.2
Not declared	19.9
USDA Choice	
Yes	9.7
Not declared	90.3
Grass-fed	
Yes	6.2
Not declared	93.8
American/USA Origin	
Yes	15.3
No	2.2
Unknown	82.5

USDA: United States Department of Agriculture

Table S6. Detection rates and concentration distributions for each of five hormones, by fat tissue and non-fat (muscle, organs, ground, etc.) tissue. Samples below the limit of detection were imputed as the limit of detection divided by the square-root of 2, prior to calculation of distribution statistics.

Tissue	HGP	LOD (pg/mg)	N samples	Percent detected (N)	Mean (pg/mg)	95th percentile (pg/mg)	Max (pg/mg)
Fat	Epitestosterone	0.1	76	36% (27)	0.20	0.61	3.09
	Melengestrol Acetate	0.1	76	25% (19)	0.65	3.25	4.07
	Progesterone	0.5	76	21% (16)	4.91	48.20	70.10
	Testosterone	0.1	76	4% (3)	0.20	0.07[a]	4.91
	Trenbolone	0.1	76	1% (1)	0.07	0.07[a]	0.16
Non-fat	Epitestosterone	0.1	321	17% (55)	0.10	0.26	1.07
	Melengestrol Acetate	0.1	321	18% (59)	0.13	0.40	2.38
	Progesterone	0.5	321	24% (77)	1.84	9.41	26.40
	Testosterone	0.1	321	6% (19)	0.09	0.15	1.38
	Trenbolone	0.1	321	0% (0)	0.07[a]	0.07[a]	0.07[a]

HGP: hormonal growth promotant; LOD: limit of detection

[a]Equivalent to the limit of detection divided by the square root of 2.

Table S7. Estimates of usual daily testosterone intake from beef consumption, across various demographic groups and intake scenarios, in a nationally representative U.S. sample from the National Health and Nutrition Examination Survey, 2015-2018.

Demographic category (age range in years)	Total testosterone intake from estimated usual daily intake[ab] of beef, µg/kg/day (Hazard index)[c]						
	Total n[d]	Eaters n[e]	Mean	Median	95th%	99th%	ADI (µg/kg/day)
Typical intake scenario[f]							
Females							
Early childhood (1-5)	798	120	0.00021 (<0.01)	0.0002 (<0.01)	0.0004 (<0.01)	0.00052 (<0.01)	2
Middle childhood (6-9)	605	84	0.00016 (<0.01)	0.00014 (<0.01)	0.0003 (<0.01)	0.0004 (<0.01)	2
Adolescence (10-19)	1430	220	0.00011 (<0.01)	0.000098 (<0.01)	0.00021 (<0.01)	0.00029 (<0.01)	2
Young-to-middle adulthood (20-54)	2883	410	0.00012 (<0.01)	0.00012 (<0.01)	0.00021 (<0.01)	0.00025 (<0.01)	2
Postmenopausal (55+)	2154	350	0.00016 (<0.01)	0.00015 (<0.01)	0.00027 (<0.01)	0.00033 (<0.01)	2
Males							
Early childhood (1-5)	823	120	0.0003 (<0.01)	0.00027 (<0.01)	0.00055 (<0.01)	0.00072 (<0.01)	2

Middle childhood (6-9)	612	94	0.00028 (<0.01)	0.00026 (<0.01)	0.00052 (<0.01)	0.00068 (<0.01)	2
Adolescence (10-19)	1428	210	0.00022 (<0.01)	0.0002 (<0.01)	0.00042 (<0.01)	0.00056 (<0.01)	2
Young-to-middle adulthood (20-54)	2576	390	0.00021 (<0.01)	0.0002 (<0.01)	0.00035 (<0.01)	0.00043 (<0.01)	2
Late adulthood (55+)	2146	340	0.00019 (<0.01)	0.00019 (<0.01)	0.00031 (<0.01)	0.00037 (<0.01)	2
High intake scenario[f]							
Females							
Early childhood (1-5)	798	120	0.00045 (<0.01)	0.00042 (<0.01)	0.00084 (<0.01)	0.0011 (<0.01)	2
Middle childhood (6-9)	605	84	0.00033 (<0.01)	0.0003 (<0.01)	0.00063 (<0.01)	0.00086 (<0.01)	2
Adolescence (10-19)	1430	220	0.00023 (<0.01)	0.0002 (<0.01)	0.00044 (<0.01)	0.0006 (<0.01)	2
Young-to-middle adulthood (20-54)	2883	410	0.00026 (<0.01)	0.00025 (<0.01)	0.00043 (<0.01)	0.00054 (<0.01)	2
Postmenopausal (55+)	2154	350	0.00033 (<0.01)	0.00032 (<0.01)	0.00056 (<0.01)	0.00068 (<0.01)	2
Males							

Early childhood (1-5)	823	120	0.00064 (<0.01)	0.00059 (<0.01)	0.0012 (<0.01)	0.0015 (<0.01)	2
Middle childhood (6-9)	612	94	0.00058 (<0.01)	0.00053 (<0.01)	0.0011 (<0.01)	0.0014 (<0.01)	2
Adolescence (10-19)	1428	210	0.00047 (<0.01)	0.00043 (<0.01)	0.00089 (<0.01)	0.0012 (<0.01)	2
Young-to-middle adulthood (20-54)	2576	390	0.00043 (<0.01)	0.00042 (<0.01)	0.00073 (<0.01)	0.00088 (<0.01)	2
Late adulthood (55+)	2146	340	0.0004 (<0.01)	0.00039 (<0.01)	0.00064 (<0.01)	0.00077 (<0.01)	2
				Max intake scenario[f]			
Females							
Early childhood (1-5)	798	120	0.0027 (<0.01)	0.0025 (<0.01)	0.005 (<0.01)	0.0066 (<0.01)	2
Middle childhood (6-9)	605	84	0.002 (<0.01)	0.0018 (<0.01)	0.0038 (<0.01)	0.0052 (<0.01)	2
Adolescence (10-19)	1430	220	0.0014 (<0.01)	0.0012 (<0.01)	0.0027 (<0.01)	0.0037 (<0.01)	2
Young-to-middle adulthood (20-54)	2883	410	0.0016 (<0.01)	0.0015 (<0.01)	0.0026 (<0.01)	0.0032 (<0.01)	2

Postmenopausal (55+)	2154	350	0.002 (<0.01)	0.0019 (<0.01)	0.0033 (<0.01)	0.0041 (<0.01)	2
Males							
Early childhood (1-5)	823	120	0.0038 (<0.01)	0.0035 (<0.01)	0.0068 (<0.01)	0.0092 (<0.01)	2
Middle childhood (6-9)	612	94	0.0035 (<0.01)	0.0032 (<0.01)	0.0064 (<0.01)	0.0083 (<0.01)	2
Adolescence (10-19)	1428	210	0.0028 (<0.01)	0.0026 (<0.01)	0.0054 (<0.01)	0.0073 (<0.01)	2
Young-to-middle adulthood (20-54)	2576	390	0.0026 (<0.01)	0.0025 (<0.01)	0.0044 (<0.01)	0.0054 (<0.01)	2
Late adulthood (55+)	2146	340	0.0024 (<0.01)	0.0023 (<0.01)	0.0039 (<0.01)	0.0048 (<0.01)	2

ADI: acceptable daily intake; NHANES: National Health and Nutrition Examination Survey; JECFA: Joint Food and Agriculture Organization of the United Nations/World Health Organization Expert Committee on Food Additives
[a]Usual daily intake is based on two non-consecutive 24-hour recalls from NHANES dietary recall data (2015-2018).
[b]National Cancer Institute method for estimating the usual daily intake of episodically consumed foods (Tooze et al. 2006; https://pubmed.ncbi.nlm.nih.gov/17000190/). Testosterone intake is the sum of testosterone and epitestosterone consumed from beef, assuming all epitestosterone is converted to testosterone.
[c]The hazard index is defined as the ratio of the intake estimate to the ADI for testosterone established by JECFA, 0-2 µg/kg/day. A hazard index greater than 1 indicates that the testosterone intake estimate exceeded the established ADI. The estimated proportion of the population exceeding the ADI is 0.
[d]Total number of NHANES sample persons in demographic category who completed the NHANES dietary questionnaire.
[e]Number of NHANES sample persons consuming beef on both of the two dietary recall days. This set of individuals is used to estimate logistic and linear mixed models for predicting usual daily intake, with a sample size of 50 or greater recommended to ensure sufficient stability of the models. Subsequently, the models are used in Monte Carlo simulations to estimate usual daily intake for all individuals (see "Total n" column),

including those not reporting beef consumption in the two 24-hour recalls conducted by NHANES, using demographic and other data to predict long-term daily intake. This approach is described in greater detail in Tooze et al. 2006 (https://pubmed.ncbi.nlm.nih.gov/17000190/).

[f]Exposure scenarios are defined by the assumed concentration of growth hormone in beef consumption reported by NHANES participants, as measured in purchased retail beef products using laboratory methods described elsewhere. "Typical" intake assumes the mean concentration was consumed; "high" intake assumes the 95th percentile was consumed; and "max" intake assumes the highest observed concentration was consumed.

Table S8. Two-day average estimates of daily melengestrol acetate intake from beef consumption, across various demographic groups and intake scenarios, in the U.S. population (NHANES 2015-2018).

Demographic category (age range in years)	Two-day average daily melengestrol acetate intake from intake[a] of beef, µg/kg/day (Hazard index)[b]						
	Total n[c]	Eaters n[d]	Mean	Median	95th%	99th%	ADI (µg/kg/day)
Typical intake scenario[e]							
Females							
Early childhood (1-5)	798	380	0.0003 (0.01)	0.00021 (0.01)	0.00094 (0.03)	0.0016 (0.05)	0.03
Middle childhood (6-9)	605	312	0.00024 (0.01)	0.00016 (0.01)	0.00074 (0.02)	0.0013 (0.04)	0.03
Adolescence (10-19)	1430	663	0.00015 (0.01)	0.0001 (<0.01)	0.00043 (0.01)	0.0008 (0.03)	0.03
Young-to-middle adulthood (20-54)	2883	1455	0.00015 (<0.01)	0.000098 (<0.01)	0.00043 (0.01)	0.00083 (0.03)	0.03
Postmenopausal (55+)	2154	1109	0.00014 (<0.01)	0.00011 (<0.01)	0.00039 (0.01)	0.00067 (0.02)	0.03
Males							
Early childhood (1-5)	823	369	0.00031 (0.01)	0.0002 (0.01)	0.00091 (0.03)	0.0019 (0.06)	0.03
Middle childhood (6-9)	612	308	0.00029 (0.01)	0.0002 (0.01)	0.001 (0.03)	0.0013 (0.04)	0.03

Adolescence (10-19)	1428	793	0.00021 (0.01)	0.00016 (0.01)	0.00059 (0.02)	0.001 (0.03)	0.03
Young-to-middle adulthood (20-54)	2576	1413	0.00019 (0.01)	0.00013 (<0.01)	0.00053 (0.02)	0.001 (0.03)	0.03
Late adulthood (55+)	2146	1229	0.00016 (0.01)	0.00012 (<0.01)	0.00041 (0.01)	0.00067 (0.02)	0.03
High intake scenario[e]							
Females							
Early childhood (1-5)	798	380	0.00094 (0.03)	0.00066 (0.02)	0.0029 (0.10)	0.0051 (0.17)	0.03
Middle childhood (6-9)	605	312	0.00075 (0.03)	0.0005 (0.02)	0.0023 (0.08)	0.0041 (0.14)	0.03
Adolescence (10-19)	1430	663	0.00047 (0.02)	0.00032 (0.01)	0.0013 (0.04)	0.0025 (0.08)	0.03
Young-to-middle adulthood (20-54)	2883	1455	0.00045 (0.02)	0.0003 (0.01)	0.0013 (0.04)	0.0026 (0.09)	0.03
Postmenopausal (55+)	2154	1109	0.00043 (0.01)	0.00033 (0.01)	0.0012 (0.04)	0.0021 (0.07)	0.03
Males							
Early childhood (1-5)	823	369	0.00097 (0.03)	0.00062 (0.02)	0.0028 (0.09)	0.006 (0.20)	0.03

Middle childhood (6-9)	612	308	0.0009 (0.03)	0.00061 (0.02)	0.0033 (0.11)	0.0041 (0.14)	0.03
Adolescence (10-19)	1428	793	0.00066 (0.02)	0.00049 (0.02)	0.0018 (0.06)	0.0032 (0.11)	0.03
Young-to-middle adulthood (20-54)	2576	1413	0.00059 (0.02)	0.00039 (0.01)	0.0016 (0.05)	0.0032 (0.11)	0.03
Late adulthood (55+)	2146	1229	0.0005 (0.02)	0.00037 (0.01)	0.0013 (0.04)	0.0021 (0.07)	0.03
Max intake scenario[e]							
Females							
Early childhood (1-5)	798	380	0.0056 (0.19)	0.0039 (0.13)	0.017 (0.58)	0.03 (1.00)	0.03
Middle childhood (6-9)	605	312	0.0044 (0.15)	0.003 (0.10)	0.014 (0.46)	0.024 (0.82)	0.03
Adolescence (10-19)	1430	663	0.0028 (0.09)	0.0019 (0.06)	0.008 (0.27)	0.015 (0.49)	0.03
Young-to-middle adulthood (20-54)	2883	1455	0.0027 (0.09)	0.0018 (0.06)	0.0079 (0.26)	0.015 (0.51)	0.03
Postmenopausal (55+)	2154	1109	0.0025 (0.08)	0.002 (0.07)	0.0073 (0.24)	0.012 (0.41)	0.03
Males							

Early childhood (1-5)	823	369	0.0058 (0.19)	0.0037 (0.12)	0.017 (0.56)	0.036 (1.19)	0.03
Middle childhood (6-9)	612	308	0.0053 (0.18)	0.0036 (0.12)	0.019 (0.65)	0.024 (0.81)	0.03
Adolescence (10-19)	1428	793	0.0039 (0.13)	0.0029 (0.10)	0.011 (0.36)	0.019 (0.63)	0.03
Young-to-middle adulthood (20-54)	2576	1413	0.0035 (0.12)	0.0023 (0.08)	0.0097 (0.32)	0.019 (0.63)	0.03
Late adulthood (55+)	2146	1229	0.003 (0.10)	0.0022 (0.07)	0.0076 (0.25)	0.012 (0.41)	0.03

ADI: acceptable daily intake; NHANES: National Health and Nutrition Examination Survey; JECFA: Joint Food and Agriculture Organization of the United Nations/World Health Organization Expert Committee on Food Additives

[a]Two-day average daily intake estimates are based on two non-consecutive 24-hour recalls from NHANES dietary recall data (2015-2018).

[b]The hazard index is defined as the ratio of the intake estimate to the ADI for melengestrol acetate established by JECFA, 0-0.03 µg/kg/day. A hazard index greater than 1 indicates that the melengestrol acetate intake estimate exceeded the established ADI. The estimated proportion of the population exceeding the ADI is 0.

[c]Total number of NHANES sample persons in demographic category who completed the NHANES dietary questionnaire.

[d]Number of NHANES sample persons responding to both 24-hour dietary recalls and consuming beef on at least one of the days. These individuals were used to estimate two-day average intake.

[e]Exposure scenarios are defined by the assumed concentration of growth hormone in beef consumption reported by NHANES participants, as measured in purchased retail beef products using laboratory methods described elsewhere. "Typical" intake assumes the mean concentration was consumed; "high" intake assumes the 95th percentile was consumed; and "max" intake assumes the highest observed concentration was consumed.

Table S9. Two-day average estimates of daily progesterone intake from beef consumption, across various demographic groups and intake scenarios, in the U.S. population (NHANES 2015-2018).

Demographic category (age range in years)	Two-day average daily progesterone intake from intake[a] of beef, µg/kg/day (Hazard index)[b]						
	Total n[c]	Eaters n[d]	Mean	Median	95th%	99th%	ADI (µg/kg/day)
Typical intake scenario[e]							
Females							
Early childhood (1-5)	798	380	0.0043 (<0.01)	0.0031 (<0.01)	0.014 (<0.01)	0.023 (<0.01)	30
Middle childhood (6-9)	605	312	0.0034 (<0.01)	0.0023 (<0.01)	0.011 (<0.01)	0.019 (<0.01)	30
Adolescence (10-19)	1430	663	0.0022 (<0.01)	0.0015 (<0.01)	0.0062 (<0.01)	0.011 (<0.01)	30
Young-to-middle adulthood (20-54)	2883	1455	0.0021 (<0.01)	0.0014 (<0.01)	0.0061 (<0.01)	0.012 (<0.01)	30
Postmenopausal (55+)	2154	1109	0.002 (<0.01)	0.0015 (<0.01)	0.0056 (<0.01)	0.0095 (<0.01)	30
Males							
Early childhood (1-5)	823	369	0.0045 (<0.01)	0.0029 (<0.01)	0.013 (<0.01)	0.028 (<0.01)	30
Middle childhood (6-9)	612	308	0.0041 (<0.01)	0.0028 (<0.01)	0.015 (<0.01)	0.019 (<0.01)	30

Adolescence (10-19)	1428	793	0.003 (<0.01)	0.0023 (<0.01)	0.0085 (<0.01)	0.015 (<0.01)	30
Young-to-middle adulthood (20-54)	2576	1413	0.0027 (<0.01)	0.0018 (<0.01)	0.0075 (<0.01)	0.015 (<0.01)	30
Late adulthood (55+)	2146	1229	0.0023 (<0.01)	0.0017 (<0.01)	0.0059 (<0.01)	0.0097 (<0.01)	30
High intake scenario[e]							
Females							
Early childhood (1-5)	798	380	0.022 (<0.01)	0.016 (<0.01)	0.069 (<0.01)	0.12 (<0.01)	30
Middle childhood (6-9)	605	312	0.018 (<0.01)	0.012 (<0.01)	0.054 (<0.01)	0.097 (<0.01)	30
Adolescence (10-19)	1430	663	0.011 (<0.01)	0.0074 (<0.01)	0.032 (<0.01)	0.059 (<0.01)	30
Young-to-middle adulthood (20-54)	2883	1455	0.011 (<0.01)	0.0071 (<0.01)	0.031 (<0.01)	0.061 (<0.01)	30
Postmenopausal (55+)	2154	1109	0.01 (<0.01)	0.0078 (<0.01)	0.029 (<0.01)	0.049 (<0.01)	30
Males							
Early childhood (1-5)	823	369	0.023 (<0.01)	0.015 (<0.01)	0.067 (<0.01)	0.14 (<0.01)	30

Middle childhood (6-9)	612	308	0.021 (<0.01)	0.014 (<0.01)	0.077 (<0.01)	0.097 (<0.01)	30
Adolescence (10-19)	1428	793	0.015 (<0.01)	0.012 (<0.01)	0.043 (<0.01)	0.075 (<0.01)	30
Young-to-middle adulthood (20-54)	2576	1413	0.014 (<0.01)	0.0092 (<0.01)	0.038 (<0.01)	0.075 (<0.01)	30
Late adulthood (55+)	2146	1229	0.012 (<0.01)	0.0088 (<0.01)	0.03 (<0.01)	0.049 (<0.01)	30
Max intake scenario[e]							
Females							
Early childhood (1-5)	798	380	0.062 (<0.01)	0.044 (<0.01)	0.19 (0.01)	0.33 (0.01)	30
Middle childhood (6-9)	605	312	0.049 (<0.01)	0.033 (<0.01)	0.15 (0.01)	0.27 (0.01)	30
Adolescence (10-19)	1430	663	0.031 (<0.01)	0.021 (<0.01)	0.089 (<0.01)	0.16 (0.01)	30
Young-to-middle adulthood (20-54)	2883	1455	0.03 (<0.01)	0.02 (<0.01)	0.088 (<0.01)	0.17 (0.01)	30
Postmenopausal (55+)	2154	1109	0.028 (<0.01)	0.022 (<0.01)	0.081 (<0.01)	0.14 (<0.01)	30
Males							

Early childhood (1-5)	823	369	0.064 (<0.01)	0.041 (<0.01)	0.19 (0.01)	0.4 (0.01)	30
Middle childhood (6-9)	612	308	0.059 (<0.01)	0.04 (<0.01)	0.22 (0.01)	0.27 (0.01)	30
Adolescence (10-19)	1428	793	0.043 (<0.01)	0.032 (<0.01)	0.12 (<0.01)	0.21 (0.01)	30
Young-to-middle adulthood (20-54)	2576	1413	0.039 (<0.01)	0.026 (<0.01)	0.11 (<0.01)	0.21 (0.01)	30
Late adulthood (55+)	2146	1229	0.033 (<0.01)	0.025 (<0.01)	0.085 (<0.01)	0.14 (<0.01)	30

ADI: acceptable daily intake; NHANES: National Health and Nutrition Examination Survey; JECFA: Joint Food and Agriculture Organization of the United Nations/World Health Organization Expert Committee on Food Additives

[a]Two-day average daily intake estimates are based on two non-consecutive 24-hour recalls from NHANES dietary recall data (2015-2018).

[b]The hazard index is defined as the ratio of the intake estimate to the ADI for progesterone established by JECFA, 0-30 μg/kg/day. A hazard index greater than 1 indicates that the progesterone intake estimate exceeded the established ADI. The estimated proportion of the population exceeding the ADI is 0.

[c]Total number of NHANES sample persons in demographic category who completed the NHANES dietary questionnaire.

[d]Number of NHANES sample persons responding to both 24-hour dietary recalls and consuming beef on at least one of the days. These individuals were used to estimate two-day average intake.

[e]Exposure scenarios are defined by the assumed concentration of growth hormone in beef consumption reported by NHANES participants, as measured in purchased retail beef products using laboratory methods described elsewhere. "Typical" intake assumes the mean concentration was consumed; "high" intake assumes the 95th percentile was consumed; and "max" intake assumes the highest observed concentration was consumed.

Table S10. Two-day average estimates of daily total testosterone intake (sum of testosterone and epitestosterone) from beef consumption, across various demographic groups and intake scenarios, in the U.S. population (NHANES 2015-2018).

Demographic category (age range in years)	Two-day average daily total testosterone (epitestosterone and testosterone)[a] intake from intake[b] of beef, µg/kg/day (Hazard index)[c]						
	Total n[d]	Eaters n[e]	Mean	Median	95th%	99th%	ADI (µg/kg/day)
Typical intake scenario[e]							
Females							
Early childhood (1-5)	798	380	0.00046 (<0.01)	0.00032 (<0.01)	0.0014 (<0.01)	0.0025 (<0.01)	2
Middle childhood (6-9)	605	312	0.00036 (<0.01)	0.00024 (<0.01)	0.0011 (<0.01)	0.002 (<0.01)	2
Adolescence (10-19)	1430	663	0.00023 (<0.01)	0.00015 (<0.01)	0.00065 (<0.01)	0.0012 (<0.01)	2
Young-to-middle adulthood (20-54)	2883	1455	0.00022 (<0.01)	0.00015 (<0.01)	0.00065 (<0.01)	0.0013 (<0.01)	2
Postmenopausal (55+)	2154	1109	0.00021 (<0.01)	0.00016 (<0.01)	0.00059 (<0.01)	0.001 (<0.01)	2
Males							
Early childhood (1-5)	823	369	0.00047 (<0.01)	0.0003 (<0.01)	0.0014 (<0.01)	0.0029 (<0.01)	2
Middle childhood (6-9)	612	308	0.00043 (<0.01)	0.0003 (<0.01)	0.0016 (<0.01)	0.002 (<0.01)	2

Adolescence (10-19)	1428	793	0.00032 (<0.01)	0.00024 (<0.01)	0.0009 (<0.01)	0.0015 (<0.01)	2
Young-to-middle adulthood (20-54)	2576	1413	0.00028 (<0.01)	0.00019 (<0.01)	0.0008 (<0.01)	0.0015 (<0.01)	2
Late adulthood (55+)	2146	1229	0.00024 (<0.01)	0.00018 (<0.01)	0.00062 (<0.01)	0.001 (<0.01)	2
High intake scenario[e]							
Females							
Early childhood (1-5)	798	380	0.00096 (<0.01)	0.00067 (<0.01)	0.003 (<0.01)	0.0051 (<0.01)	2
Middle childhood (6-9)	605	312	0.00076 (<0.01)	0.00051 (<0.01)	0.0023 (<0.01)	0.0042 (<0.01)	2
Adolescence (10-19)	1430	663	0.00048 (<0.01)	0.00032 (<0.01)	0.0014 (<0.01)	0.0025 (<0.01)	2
Young-to-middle adulthood (20-54)	2883	1455	0.00046 (<0.01)	0.00031 (<0.01)	0.0014 (<0.01)	0.0026 (<0.01)	2
Postmenopausal (55+)	2154	1109	0.00044 (<0.01)	0.00034 (<0.01)	0.0012 (<0.01)	0.0021 (<0.01)	2
Males							
Early childhood (1-5)	823	369	0.00099 (<0.01)	0.00063 (<0.01)	0.0029 (<0.01)	0.0061 (<0.01)	2

Middle childhood (6-9)	612	308	0.00091 (<0.01)	0.00062 (<0.01)	0.0033 (<0.01)	0.0042 (<0.01)	2
Adolescence (10-19)	1428	793	0.00067 (<0.01)	0.0005 (<0.01)	0.0019 (<0.01)	0.0032 (<0.01)	2
Young-to-middle adulthood (20-54)	2576	1413	0.00059 (<0.01)	0.0004 (<0.01)	0.0017 (<0.01)	0.0032 (<0.01)	2
Late adulthood (55+)	2146	1229	0.00051 (<0.01)	0.00038 (<0.01)	0.0013 (<0.01)	0.0021 (<0.01)	2
Max intake scenario[e]							
Females							
Early childhood (1-5)	798	380	0.0058 (<0.01)	0.0041 (<0.01)	0.018 (0.01)	0.031 (0.02)	2
Middle childhood (6-9)	605	312	0.0046 (<0.01)	0.0031 (<0.01)	0.014 (0.01)	0.025 (0.01)	2
Adolescence (10-19)	1430	663	0.0029 (<0.01)	0.0019 (<0.01)	0.0082 (<0.01)	0.015 (0.01)	2
Young-to-middle adulthood (20-54)	2883	1455	0.0028 (<0.01)	0.0019 (<0.01)	0.0081 (<0.01)	0.016 (0.01)	2
Postmenopausal (55+)	2154	1109	0.0026 (<0.01)	0.002 (<0.01)	0.0075 (<0.01)	0.013 (0.01)	2
Males							

Early childhood (1-5)	823	369	0.0059 (<0.01)	0.0038 (<0.01)	0.017 (0.01)	0.037 (0.02)	2
Middle childhood (6-9)	612	308	0.0055 (<0.01)	0.0037 (<0.01)	0.02 (0.01)	0.025 (0.01)	2
Adolescence (10-19)	1428	793	0.004 (<0.01)	0.003 (<0.01)	0.011 (0.01)	0.019 (0.01)	2
Young-to-middle adulthood (20-54)	2576	1413	0.0036 (<0.01)	0.0024 (<0.01)	0.01 (0.01)	0.019 (0.01)	2
Late adulthood (55+)	2146	1229	0.0031 (<0.01)	0.0023 (<0.01)	0.0078 (<0.01)	0.013 (0.01)	2

ADI: acceptable daily intake; NHANES: National Health and Nutrition Examination Survey; JECFA: Joint Food and Agriculture Organization of the United Nations/World Health Organization Expert Committee on Food Additives

[a]Two-day average daily intake estimates are based on two non-consecutive 24-hour recalls from NHANES dietary recall data (2015-2018). Testosterone intake is the sum of testosterone and epitestosterone consumed from beef, assuming all epitestosterone is converted to testosterone.

[b]The hazard index is defined as the ratio of the intake estimate to the ADI for testosterone established by JECFA, 0-2 g/kg/day. A hazard index greater than 1 indicates that the testosterone intake estimate exceeded the established ADI. The estimated proportion of the population exceeding the ADI is 0.

[c]Total number of NHANES sample persons in demographic category who completed the NHANES dietary questionnaire.

[d]Number of NHANES sample persons responding to both 24-hour dietary recalls and consuming beef on at least one of the days. These individuals were used to estimate two-day average intake.

[e]Exposure scenarios are defined by the assumed concentration of growth hormone in beef consumption reported by NHANES participants, as measured in purchased retail beef products using laboratory methods described elsewhere. "Typical" intake assumes the mean concentration was consumed; "high" intake assumes the 95th percentile was consumed; and "max" intake assumes the highest observed concentration was consumed.
